# Projected asymmetric response of Adélie penguins to Antarctic climate change

**DOI:** 10.1038/srep28785

**Published:** 2016-06-29

**Authors:** Megan A. Cimino, Heather J. Lynch, Vincent S. Saba, Matthew J. Oliver

**Affiliations:** 1 College of Earth Ocean and Environment, University of Delaware, 700 Pilottown Rd., Lewes, DE 19958, United States; 2 Stony Brook University, 113 Life Sciences Bldg., Stony Brook, NY 11794, United States; 3 NOAA National Marine Fisheries Service, Northeast Fisheries Science Center, c/o Geophysical Fluid Dynamics Laboratory, Princeton University Forrestal Campus, 201 Forrestal Road, Princeton, NJ 08540, United States

## Abstract

The contribution of climate change to shifts in a species’ geographic distribution is a critical and often unresolved ecological question. Climate change in Antarctica is asymmetric, with cooling in parts of the continent and warming along the West Antarctic Peninsula (WAP). The Adélie penguin (*Pygoscelis adeliae*) is a circumpolar meso-predator exposed to the full range of Antarctic climate and is undergoing dramatic population shifts coincident with climate change. We used true presence-absence data on Adélie penguin breeding colonies to estimate past and future changes in habitat suitability during the chick-rearing period based on historic satellite observations and future climate model projections. During the contemporary period, declining Adélie penguin populations experienced more years with warm sea surface temperature compared to populations that are increasing. Based on this relationship, we project that one-third of current Adélie penguin colonies, representing ~20% of their current population, may be in decline by 2060. However, climate model projections suggest refugia may exist in continental Antarctica beyond 2099, buffering species-wide declines. Climate change impacts on penguins in the Antarctic will likely be highly site specific based on regional climate trends, and a southward contraction in the range of Adélie penguins is likely over the next century.

Climate has influenced the distribution patterns of penguins for millions of years^1^. The Adélie penguin has intermittently occupied the West Antarctic Peninsula (WAP) for ~6,000 years^2^, East Antarctica for at least 14,000 years^3^, and the Ross Sea for ~45,000 years^4^. The paleoclimate record suggests that periodic colony abandonment corresponds to glacial expansion or extensive permanent sea ice while reoccupation corresponds to warming periods marked by deglaciation or sea ice declines that allow access to rocky nesting habitats^2^,^3^,^4^. On the other hand, late 20^th^ century climate warming along the WAP is coincident with Adélie population declines^5^ while stable or cooling conditions around the remainder of the continent generally correspond to stable or increasing populations^6^ (Fig. 1). The contrasting effects of warming on different timescales highlights that the effect of environmental drivers on penguin distributions are relative to the climate regime under consideration.

Climate change can have profound effects on penguins’ terrestrial nesting sites and marine food resources. Adélie penguins construct nests on ice- and snow-free terrain with pebbles to keep eggs and chicks dry and out of any water. Precipitation and snowmelt can cause nest site flooding that drowns eggs and small chicks^7^, influences chick mass and survival^8^, and has been linked to population declines^9^. Tight coupling in the Antarctic food web, where sea ice, wind, and water column stability impact phytoplankton and Antarctic krill (*Euphausia superba*) recruitment^10^, has lead to concerns that climate change will cause a decrease in krill abundance, a major food source for Adélie penguins. However, recent studies show no long-term (1993–2013) change in WAP krill biomass^11^ and current krill biomass should support Adélie penguin breeding colonies^12^. Although changes in, or competition for, krill may not be the main driver of WAP population declines^13^, we cannot discount the negative effects of warm waters on krill quality^14^, or the availability of krill to penguins. Antarctic silverfish (*Pleuragramma antarcticum*), another component of Adélie penguin’s diet, have declined coincident with WAP climate changes^14^,^15^ and could also influence penguin demographics such as breeding success, chick mass and survival.

Multi-decadal satellite observations provide continuous spatiotemporal measurements of sea surface temperature (SST) and sea ice concentration (SIC) in the Southern Ocean, which are essential for a continental-scale view of Adélie penguin chick-rearing habitats^16^. The high covariance between atmospheric (e.g., air temperature, wind, sea level pressure) and oceanic conditions^17^ suggest that SST and SIC may be good proxies for the quality of penguins’ terrestrial nesting habitats that can be impacted by weather, and/or indicators of food resources. Here, we develop austral-summer chick-rearing habitat suitability (CRHS) models that simulate the quality of coastal habitats using true presence-absence Adélie penguin colony locations (Fig. 1) and satellite-derived information on SIC, SST, and bare-rock locations. We used this model to project the quality of penguin habitats in the future using global climate models assessed by the Intergovernmental Panel on Climate Change (IPCC) and a prototype, high-resolution global climate model developed by the Geophysical Fluid Dynamics Laboratory (GFDL). To understand the sensitivity of our results, we compared two species distribution models (MaxEnt and Generalized Additive Models [GAMs]) and used presence and absence data from different portions of the Adélie penguin’s range.

## Results

Adélie penguin CRHS models show the spatial distribution of novel climate and changes in CRHS compared to historic observations (Fig. 2). All CRHS models performed well (area under the curve (AUC) > 0.85) and confirmed the importance of SST, SIC and bare rock to penguin chick-rearing habitats (see Supplemental Results). In this study, novel climate is SST or SIC outside the range of average SST and SIC observations from 1978–1984 (−2.42 > SST > 1.55 °C, SIC > 95.41%), which corresponds to CRHS model training data (see methods). Interestingly, ~1.5 °C was roughly the warmest SST that increasing Adélie penguin populations experienced throughout the entire satellite record while many decreasing populations experienced SST warmer than this threshold (Supplemental Fig. 1). The WAP experienced the greatest number of novel climate years, with up to seven years of novel climate from 1981–2010 and over 40 years of novel climate using an ensemble of global climate model projections from 2011–2099 (Fig. 2a, see Supplemental Figs 2–9 for individual climate model output and Supplemental Fig. 10 for variability). Marguerite Bay appears to be on a slower warming trajectory compared to the WAP (Fig. 2a), which is also evident in the high-resolution GFDL-CM2.6 projections (Supplemental Fig. 9). While both the WAP and Ross Sea regions have been characterized by high CRHS in the recent past (Fig. 2b), our model projects a substantial decrease in CRHS along the WAP and an increase in CRHS in the Ross Sea over the next century (Fig. 2c). The Cape Adare region, home to the earliest known occupation and the largest Adélie colony^4^, had no novel climate and high CRHS in the past and future (Fig. 2a,b). CRHS in the Cape Adare region is also projected to increase in the future (Fig. 2c, Supplemental Fig. 11). At Cape Adare, SST is projected to increase from about −1 °C to 0 °C and SIC is projected to decrease from ~20% to ~10% by 2100 (Supplemental Fig. 11), with these changes in SST and SIC shifting towards peak suitability (see response curves, Supplemental Fig. 12). The northeastern Antarctic Peninsula appears to be a more favorable environment than the southwestern Antarctic Peninsula (Fig. 2a,b). From 1981–2010, the South Shetland Islands and the WAP had a similar number of novel climate years and mean CRHS (Fig. 2a,b) but CRHS improved in the South Shetland Islands (Fig. 2c). During this time, the more northerly South Sandwich and South Orkney Islands experienced no years with novel climate, higher mean CRHS and improved CRHS compared to southerly islands and the WAP. Comparing two species distribution modeling approaches and two spatial subsets of the presence-absence data, we found the modeling methods (MaxEnt vs. GAMs) produced similar results but varied more when presence or absence data was incomplete. Model output was most sensitive to incomplete absence data, especially when true presence data was included, perhaps because absence data was missing within a specific environmental niche and Adélie penguins do not occupy all available habitats (Supplemental Results).

We determined whether each penguin colony experienced novel climate from 1981 to 2010, and tested for significant differences between the number of years with novel climate at decreasing, increasing and stable populations (Fig. 3). Over 50% of decreasing penguin colonies experienced novel climate as a result of warm SST, however, no declining continental colonies experienced this novel warm SST (Fig. 3a). Only ~15% of increasing and stable populations had novel climate related to warm SST (stable n = 1, increasing n = 3), cool SST (stable n = 3, increasing n = 2), or high SIC (stable n = 4, increasing n = 1). Almost 25% of populations with an unknown trend had novel climate due to warm SST and are located along the WAP or northerly islands, suggesting these population may be in decline (Fig. 3a). Colonies with decreasing populations experienced significantly more years with novel climate than populations that were increasing in abundance (nonparametric Krustal-Wallis test and multiple comparisons test after Krustal-Wallis, p < 0.05; Fig. 3b), suggesting recent warming effects are detrimental to Adélie penguin populations. Penguin colonies near Palmer Station, which have declined by 80% since the 1970s^5^, experienced the most novel climate years (seven) (Fig. 3b).

About 47% of declining populations did not experience novel climate (Fig. 2) as measured by the environmental variables considered. It is possible that Adélie penguins are sensitive to climate that is not considered to be novel (Supplemental Fig. 1). In addition, our parsimonious model could be missing an important component of habitat suitability or there could be errors in the population trends. Because individual colonies may be impacted by idiosyncratic factors operating on fine-scales, independent of large-scale climatic influences, patterns of occupancy and trend at regional and continental-scales are required to understand the role of changing climate on habitat suitability. Therefore, additional factors, either at finer scales than captured by our datasets, or factors not included in our analysis, likely contribute to population declines. Other factors that influence population trends that we could not account for include predation^18^, competition^19^, wintering habitat^20^, weather impacts on nest sites with specific geomorphology^9^, and human impacts including tourism, pollution and fishing^19^,^21^,^22^.

Climate projections from global models assessed by the IPCC and from GFDL’s high-resolution CM2.6 reveal similar trends in the cumulative number of years with novel climate in various Antarctic sectors (Fig. 4). The southern WAP, islands, and northern WAP regions, which are already experiencing population declines, are projected to experience the greatest frequency of novel climate in this century due to warm SST (Fig. 4, see Supplemental Figs 13–20 for colony projections from each climate model). Adélie penguin colonies in East Antarctica and Enderby Land are projected to experience <10 years of novel climate by 2099 while colonies in the Ross and Amundsen Seas are projected to experience <5 years of novel climate. Model projections show the majority of future novel climate will be related to warm SST (Supplemental Figs 13–20). However, ACCESS1-0, CESM1-BGC, CMCC-CC and GFDL-CM2.6 projected a few locations in the Amundsen Sea and continental Antarctica will experience novel climate due to cool SST or high sea ice (Supplemental Figs 13,15,17,20). The magnitude of novel SST is projected to increase over time with different sectors around Antarctica changing at various rates (Supplemental Fig. 21). While novel SST was generally within ~0.5 °C above or below novel SST during the satellite record (1981–2010), SST may warm to more than 5 °C (i.e. >3 °C warmer than the contemporary period) (Supplemental Fig. 22). These large changes are projected at northern latitudes, especially the southern WAP but also in the northern WAP and islands (Supplemental Fig. 21). In the Amundsen Sea, Ross Sea, East Antarctica and Enderby Land, climate model projections suggest the distribution of SST will shift towards warmer conditions but SST will generally remain within non-novel conditions, which resulted in fewer cumulative degrees of novel climate compared to more northerly sectors (Supplemental Fig. 21).

We determined the rate that novel climate will occur at Adélie penguin colonies using satellite observations (1981–2010) and the global climate model ensemble (2011–2099) (Fig. 5). During 1981 to 2010, decreasing populations had a minimum of one, an average of four, and a maximum of seven years of novel climate over 30 years of observation (Fig. 2) illustrating differential sensitivity of colonies to novel climate. This corresponds to a rate of 3.3% to 23.3% (mean 13.3%) of novel climate per annum associated with colony decline. By 2060, our projections suggest 58% to 25% (mean 36%) of colonies could experience population declines due to novel climate alone, containing 43% to 9% (mean 21%) of the currently known Adélie penguin abundance, respectively. Similarly, by 2099, our projections suggest 78% to 51% (mean 58%) of colonies could experience declines, containing 64% to 39% (mean 46%) of the current abundance.

## Discussion

On geologic timescales, Adélie penguin populations were positively affected by warming and negatively affected by cooling^2^,^3^,^4^ but the rapid response of penguin populations to multi-decadal warming events (similar to population shifts from 1980–2010, Fig. 1) cannot be assessed in the geologic record because of its coarse temporal frequency. Our study suggests that in many regions of Antarctica climate warming has tipped past peak suitability so that further warming is no longer beneficial to Adélie penguins. Warm SSTs may drive a substantial decline in the suitability of chick-rearing habitats at northerly latitudes but several refugia, particularly in the more stable Ross and Amundsen Seas, may buffer species-wide declines under climate change projections. In the northern Ross Sea, the Cape Adare region is thought to have been a glacial refuge in the past^3^,^4^,^23^ and is projected to provide refuge in the future even as conditions warm. Most of the Cape Adare colony breeds on a large beach that is just above sea level while fewer penguins breed on the escarpment and upper terrace. Therefore, this location will only be a refuge for a large population in glacial conditions when sea level is lower. Interestingly, the northern South Sandwich and Orkney Islands appear to be more favorable chick-rearing habitats, with fewer years of novel climate, than southern islands and the WAP. WAP Adélie penguin populations may have undergone similar population boom and busts in the geologic past because the current rate of warming, while highly unusual, is not unprecedented^24^. Over the past two millennia, the WAP had high climate variability^24^ that could makes it an unstable chick-rearing site for Adélie penguins with more sporadic occupancy than seen elsewhere. The WAP appears disproportionately vulnerable to projected climate change compared to other regions. Contemporary warming on the WAP is related to both atmospheric changes^25^ and the delivery of warmer upper circumpolar deep water onto the continental shelf 26. The direct or indirect effects of this warming are detrimental to Adélie penguin populations and could lead to population declines at ~30% of colonies by 2060 and ~60% of colonies by 2099 (Fig. 5). With continued warming, new bare rock nesting habitats may become available as glaciers disintegrate but colonization will likely be limited to southern localities^27^.

Many Adélie penguin population declines were associated with novel climate due to warm SSTs, which may be associated with inadequate food resources or weather that impacts the quality of nest sites. For example, reduced prey availability or quality may inhibit penguins from meeting their energetic demands^14^ while blizzards and unprecedented snow accumulation in West Antarctica^28^ can have catastrophic impacts on penguins^15^,^29^. Adélie penguins are considered to be highly philopatric^30^, but there is surprisingly little genetic differentiation among Adélie penguin populations at the continental scale^31^. It is unknown if warming prompts Adélie penguin emigration, but repeated colony abandonment and recolonization over millennia confirm this possibility. In this study, we suggest climate novelty is detrimental to Adélie penguins. While we cannot yet establish the specific mechanism for this relationship, our study focuses attention on areas where climate change is likely to create a high frequency of unsuitable conditions during the 21^st^ century and, by contrast, suggests several refugia are likely to persist.

## Methods

### Adélie penguin colony information

**Figure 1:**
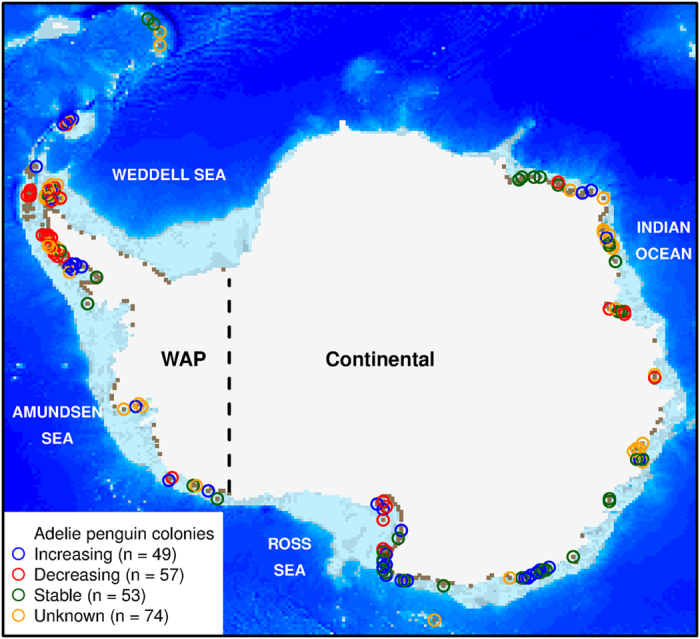
Adélie penguin breeding colonies and population status across Antarctica. Each colored circle represents a colonies’ current population trend. The black dashed line separates West Antarctic Peninsula (WAP) from continental Adélie penguin colonies. Bare rock (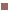) locations around the coastline and light to dark blue represents shallow to deep bathymetry modified from Cimino *et al*.^16^. The map was produced in R version 3.1.3 (www.r-project.org).

**Figure 2:**
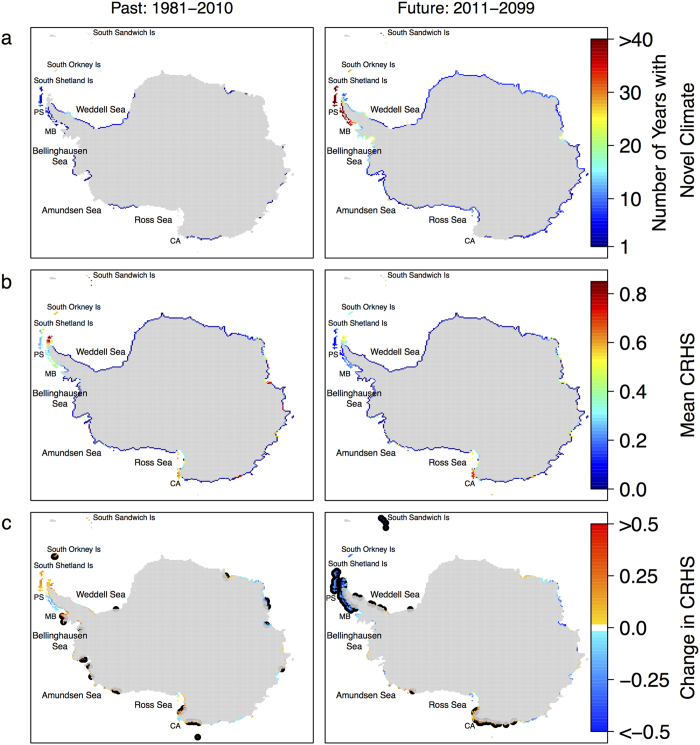
Novel climate and Adélie penguin habitat suitability during the chick-rearing period (CRHS) from past satellite observations and in the future using an ensemble of global climate models. (**a**) The number of years with novel climate, which is data outside the range of the model training data, in the past and future. (**b**) Mean CRHS and (**c**) trends in CRHS in the past and future (See Supplemental Figs 2–9 individual climate models). In the past, black outlines represent significant changes over time (p < 0.05) and in the future, black outlines show regions where all climate models project changes in the same direction. PS = Palmer Station, MB = Marguerite Bay, CA = Cape Adare. The maps were produced in R version 3.1.3 (www.r-project.org).

**Figure 3:**
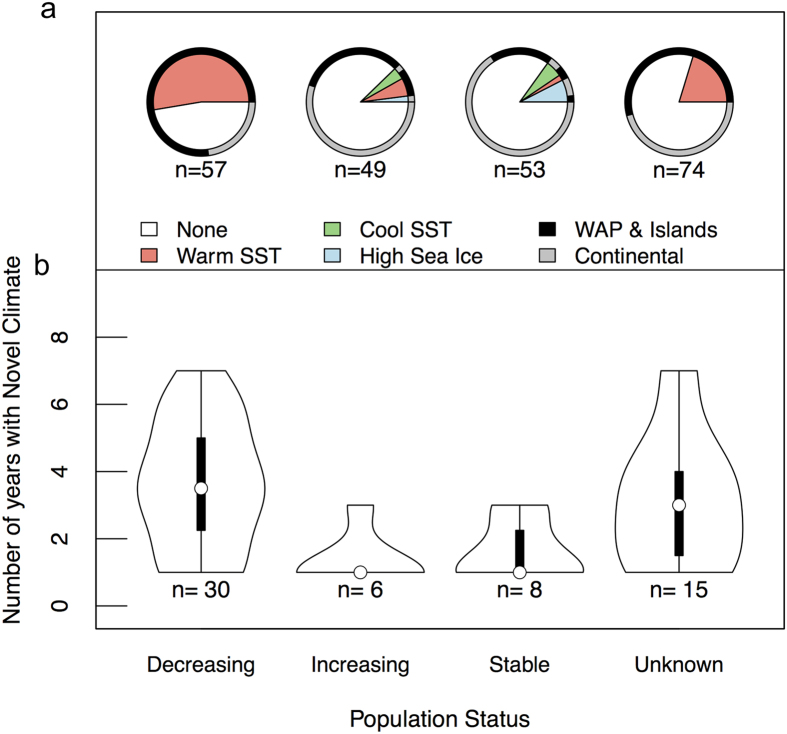
Proportion of colonies with novel climate and number of years with novel climate for each population status from 1981–2010. (**a**) The proportion of colonies for each population status that have no novel climate (none) or the leading cause of novel climate is due to warm sea surface temperature (SST), cool SST or high sea ice concentration. Colony locations were defined as WAP and Islands or Continental to understand the spatial distribution of novel climate as it relates to population trends. (**b)** Probability density of the number of years with novel climate at colonies that experienced at least one year of novel climate for populations that are decreasing, increasing, stable and unknown. The thick black box represents the interquartile range, thinner black lines represent upper and lower adjacent values and the white points are the median.

**Figure 4:**
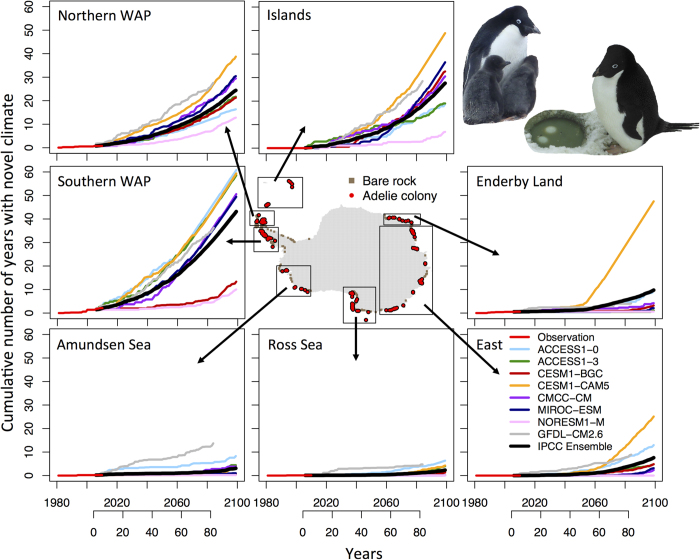
The cumulative number of years with novel climate at Adélie penguin colonies in different Antarctic sectors. Location map of Adélie penguin colonies and bare rock locations. Each line represents the mean of all colonies in that sector colored by the climate model used for the projection. Satellite observations of novel climate are in red from 1981–2010, IPCC models (RCP 8.5) from 2006–2099 and GFDL-CM2.6 from year 1–79 where atmospheric CO_2_ increases by 1% per year and atmospheric CO_2_ doubles at year 70 in the model simulation. The IPCC ensemble mean shows the average trend for all IPCC models in a sector (not including GFDL-CM2.6). See Supplemental Figs 13–20 for individual models. The map was produced in R version 3.1.3 (www.r-project.org).

**Figure 5:**
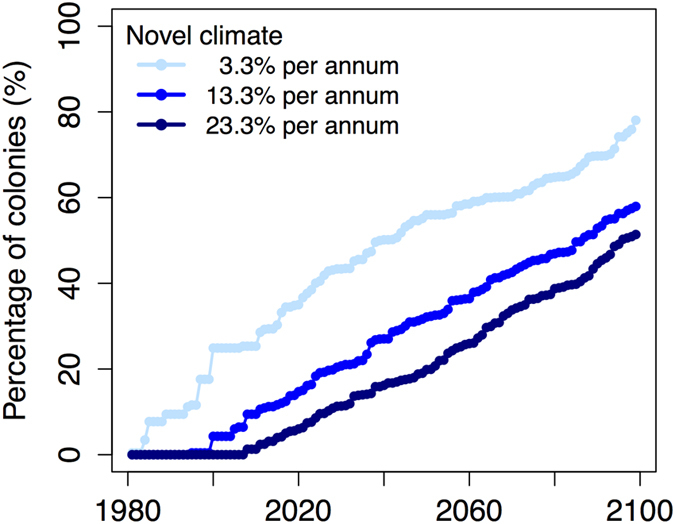
Percent of Adélie penguin breeding colonies experiencing different rates of novel climate, accounting for different colony sensitivity. Each line is the average of satellite observations from 1981–2010 and the global climate model ensemble from 2011–2099.

We obtained Adélie penguin breeding colony abundance, distribution, and population trends^6^,^32^. These datasets provided true presence and true absence locations throughout the entire Adélie penguin breeding range (Fig. 1). We considered present locations to be all colonies present in the late 70s/early 80s that had a colony status of present or presumed present. All colonies we considered to be present were mapped in Fig. 1 with the associated population status. Obtaining direct population counts for all known Adélie populations across decades has generally been constrained by logistics and the remoteness of some colonies. Lynch and LaRue (2014) reported a crude qualitative categorization of trends but more sophisticated models to estimate population trends are currently being developed as part of the Mapping Application for Penguin Populations and Projected Dynamics (MAPPPD; penguinmap.org). However, the agreement between population trends on a regional scale provides confidence in individual colony trends^32^. Penguin population trends are independent of model development (see below) and were only used to evaluate model predictions. All analyses and statistics were carried out in R (www.r-project.org, R Development Core Team, 2004).

### Environmental variables

We used satellite-derived SIC, SST, and bare-rock locations to estimate the suitability of penguin chick-rearing habitats in Antarctica (See Supplemental Methods). Years represent the austral summer field season, e.g. 1978 = Dec 1978–Feb 1979.

Historical and future climate projections of SST and SIC were obtained from the IPCC’s fifth assessment (Representative Concentration Pathway 8.5) via the Coupled Model Intercomparison Project Phase 5 (CMIP5) archive and two additional NOAA GFDL global climate models (see Supplemental Methods, Supplemental Table 1; more information on the global climate models can be found at http://cmip-pcmdi.llnl.gov/cmip5/).

Satellite-derived and climate model projections of SST and SIC are available on different spatial scales and were interpolated to a 25 × 25 km polar stereographic grid surrounding Antarctica^16^. Yearly climatologies were created for each parameter for the austral summer chick-rearing period from December to February (DJF). To understand, compare and account for climate model biases, we computed differences in the mean and standard deviation between DJF satellite observations (1978–2004) and climate model historical runs, using the historical runs from the CMIP5 models (1978–2004) and from the GFDL 1990 control simulations (years 101–140, global atmospheric CO_2_ was fixed at year 1990 concentration; CM2.1, CM2.6). We created climate model deltas by subtracting the mean of historical runs from the CMIP5 models (1978–2004) and the control simulation for GFDL models from their respective climate model projection for each year. The projections from GFDL models CM2.1 and CM2.6 were a transient climate response experiment such that CO_2_ in the atmosphere increased by 1% per year until it doubled by year 70. Further details on these models and experiments can be found in^33^. Using the delta approach to bias correct the mean, we added climate model SST and SIC deltas to the satellite climatologies to create annual climatologies of climate model conditions. Due to poor coastal resolution, we interpolated climate model projections to land (except for GFDL-CM2.6 which has high coastal resolution) and evaluated model biases based on the 200-km surrounding Antarctica and sub-Antarctic islands. From SST and SIC mean biases (Supplemental Fig. 23), we considered the best models to have average projections within ±1 °C and ±20% SIC. All models had a relatively low bias in SIC and SST variability (Supplemental Fig. 24). This resulted in seven global climate models and we also used GFDL-CM2.6 because it is a high-resolution model, which may more accurately represent dynamics in the coastal ocean. Climate model projections had a similar overall range and mean rate of change in SST and SIC (Supplemental Fig. 22).

Our study focused on the coastline around sub-Antarctic islands and Antarctica. Each coastline pixel was classified as having bare-rock present or absent. For the satellite record and climate projections, we spatially averaged SIC and SST within 75 km of each coastline pixel for each year to incorporate the ocean environment within the penguins foraging range.

### Models for penguin habitat suitability during the chick-rearing period

We modeled the suitability of Adélie penguin chick-rearing habitats using two species distribution models, a maximum entropy approach (MaxEnt) and generalized additive models (GAMs, see Supplemental Methods). Both approaches estimate the habitat suitability, ranging from 0 (least suitable) to 1 (most suitable). The CRHS models were trained with bare rock locations, and average SST and SIC climatologies from 1978–1984^16^. This multiyear average smoothed the high interannual variability characteristic of Antarctic marine environments and includes data prior to the regime shift occurring in the mid to late 1980s^34^. The model trained on 1978–1984 was then projected onto annual climatologies for SIC, SST, and bare-rock locations from 1981 to 2010. Bare rock locations were static for all years. Our approach does not account for possible changes in bare-rock locations due to sea level rise, or coastal glacial collapse exposing new nest sites. Global projections suggest the sea level could rise by 0.57–1.31 m by 2100^35^ while a higher resolution model suggests Antarctica has the potential to contribute more than a meter of sea level rise by 2100^36^. Uncertainties in annual sea level rise and glacial collapse projections inhibit us from including this into our habitat suitability model. However, while bare rock is necessary for Adélie penguin nest sites, it does not appear to be a limiting factor, as there is currently ample uncolonized bare rock in all regions of Antarctica. Our suitability projections could be overly optimistic if sea level rise inundates nesting habitat more quickly than new nesting sites are exposed due to glacial retreat, or our projections could be overly pessimistic if new nest sites are exposed by glacial retreat at a faster rate than sea level rise.

We compared models trained on different combinations of all presence-absence and only continental presence-absence data (Fig. 1) to compare model performance and spatial variability in CRHS (Supplemental Fig. 25). We separated WAP and continental locations based on opposite climate trends in the two regions (as done by^16^). We also projected the model onto future climate scenarios from our eight best performing models using GAMs and all presence-absence locations. We created CRHS maps for each year and used linear regression across time to identify locations of significant change in CRHS. We also identified areas with novel climate using multivariate environmental similarity surfaces in MaxEnt, which measures the similarity between the environment in the model training dataset and the new environment in the projection years.

## Additional Information

**How to cite this article**: Cimino, M. A. *et al*. Projected asymmetric response of Adélie penguins to Antarctic climate change. *Sci. Rep.* **6**, 28785; doi: 10.1038/srep28785 (2016).

## Supplementary Material

Supplementary Information
