# Supplementary material for: Projected asymmetric response of Adélie penguins to Antarctic climate change
Source: Sci Rep. 2016 Jun 29;6:28785. doi: 10.1038/srep28785 (PMC4926113; doi:10.1038/srep28785)
Supplement: Supplementary Information [file srep28785-s1.pdf]

Projected asymmetric response of Adélie penguins to Antarctic climate change
Megan A. Cimino, Heather J. Lynch, Vincent S. Saba, Matthew J. Oliver

Supplemental Figures

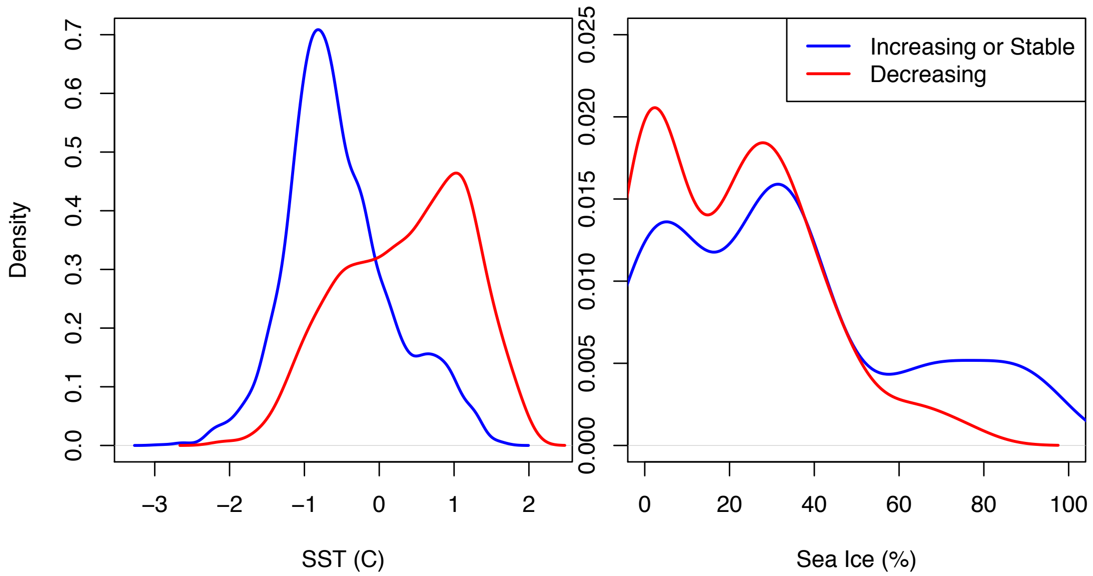

Supplemental Figure 1. The distribution of sea surface temperature (SST) and sea ice concentration at increasing or stable and decreasing Adélie penguin colonies from 1981-2010.

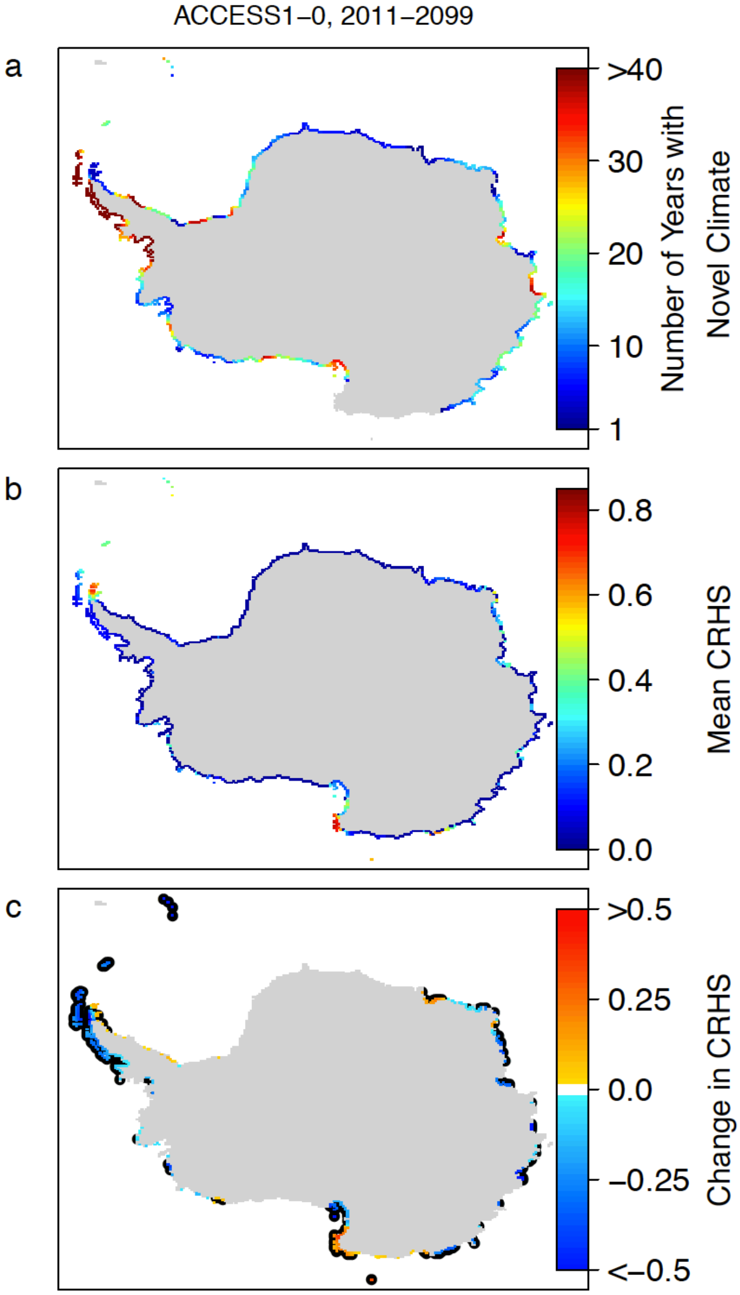

Supplemental Figure 2. Novel climate and Adélie penguin chick-rearing habitat suitability (CRHS) according to ACCESS1-0. (**a**) The number of years with novel climate, (**b**) mean CRHS and (**c**) trends in CRHS from General Additive Models using all presence-absence data from 2011-2099. Black outlines represent significant changes over time ($p < 0.05$). The maps were produced in R version 3.1.3 (www.r-project.org).

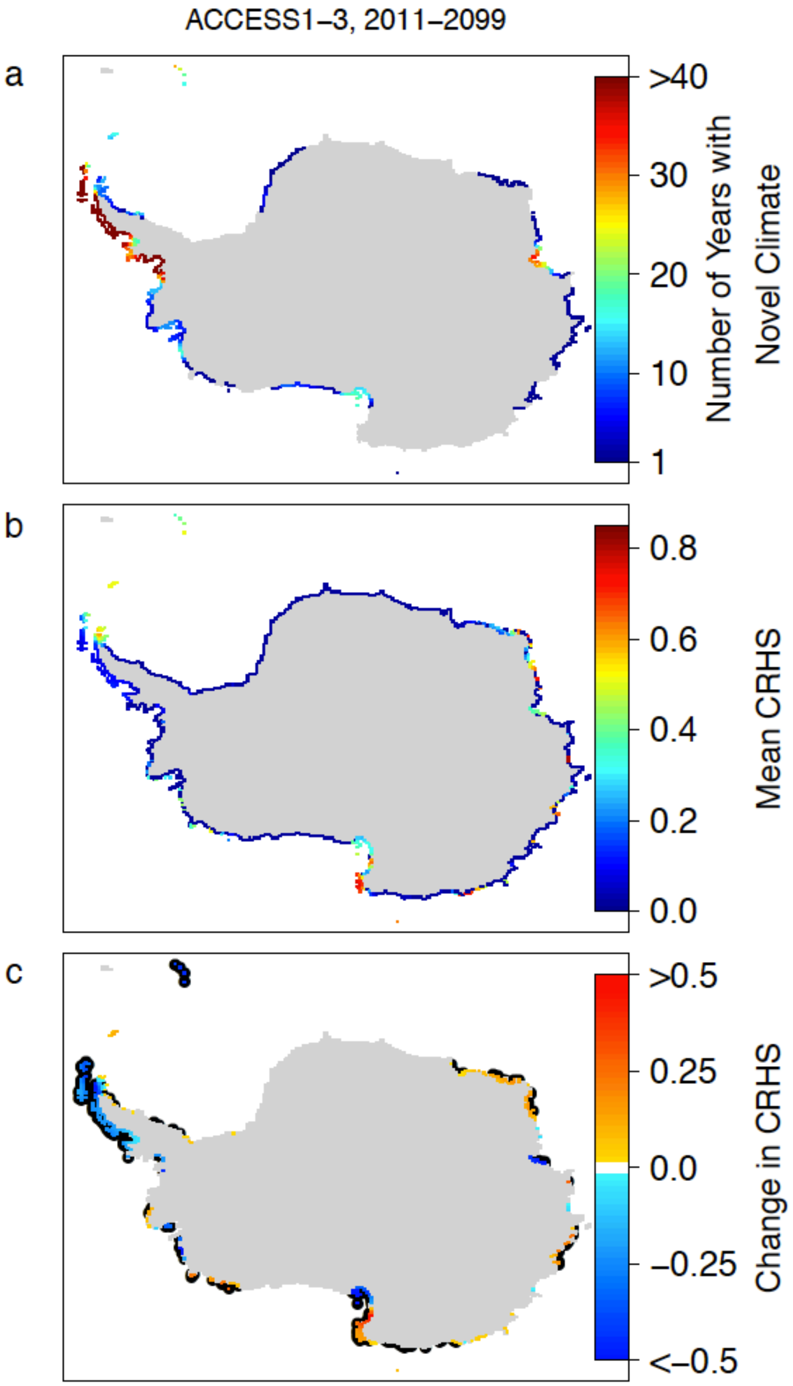

Supplemental Figure 3. Novel climate and Adélie penguin chick-rearing habitat suitability (CRHS) according to ACCESS1-3. (**a**) The number of years with novel climate, (**b**) mean CRHS and (**c**) trends in CRHS from General Additive Models using all presence-absence data from 2011-2099. Black outlines represent significant changes over time ($p < 0.05$). The maps were produced in R version 3.1.3 (www.r-project.org).

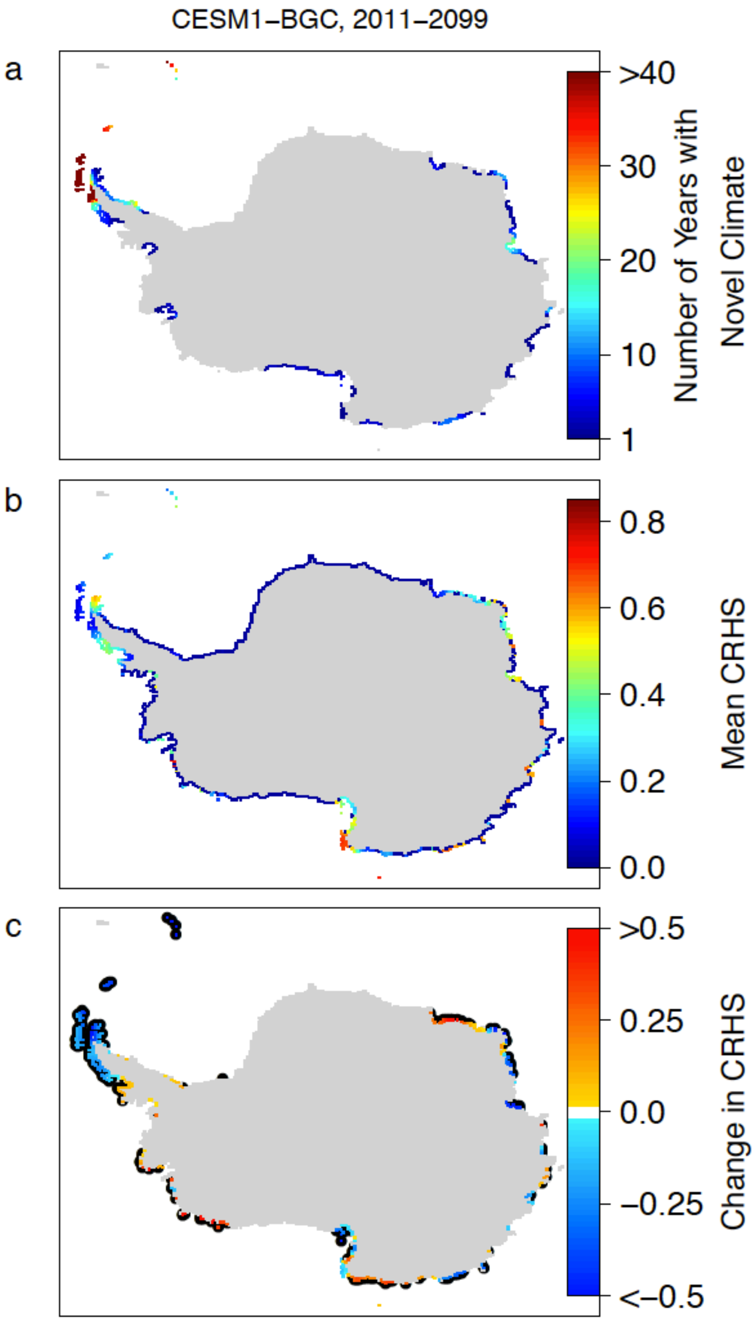

Supplemental Figure 4. Novel climate and Adélie penguin chick-rearing habitat suitability (CRHS) according to CESM1-BGC. (**a**) The number of years with novel climate, (**b**) mean CRHS and (**c**) trends in CRHS from General Additive Models using all presence-absence data from 2011-2099. Black outlines represent significant changes over time ($p < 0.05$). The maps were produced in R version 3.1.3 (www.r-project.org).

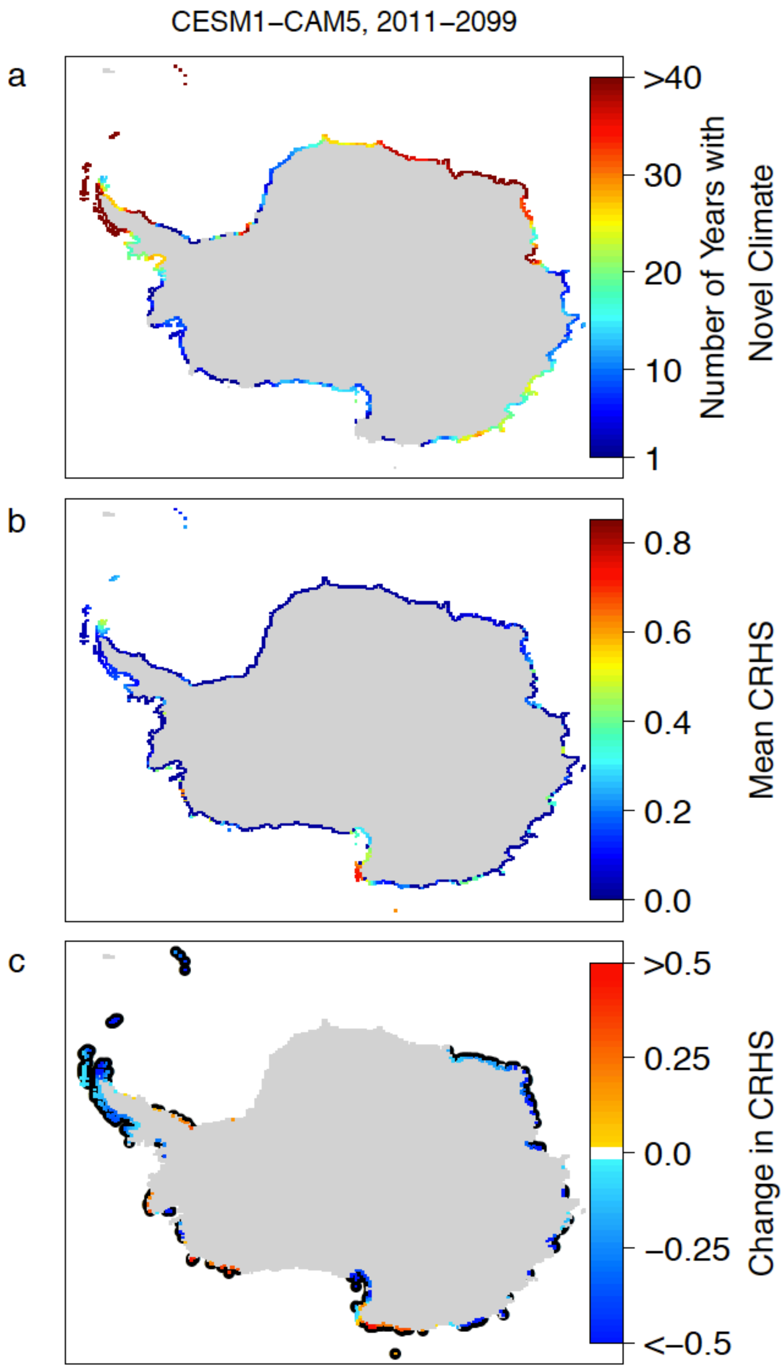

Supplemental Figure 5. Novel climate and Adélie penguin chick-rearing habitat suitability (CRHS) according to CESM1-CAM5. (**a**) The number of years with novel climate, (**b**) mean CRHS and (**c**) trends in CRHS from General Additive Models using all presence-absence data from 2011-2099. Black outlines represent significant changes over time ($p < 0.05$). The maps were produced in R version 3.1.3 (www.r-project.org).

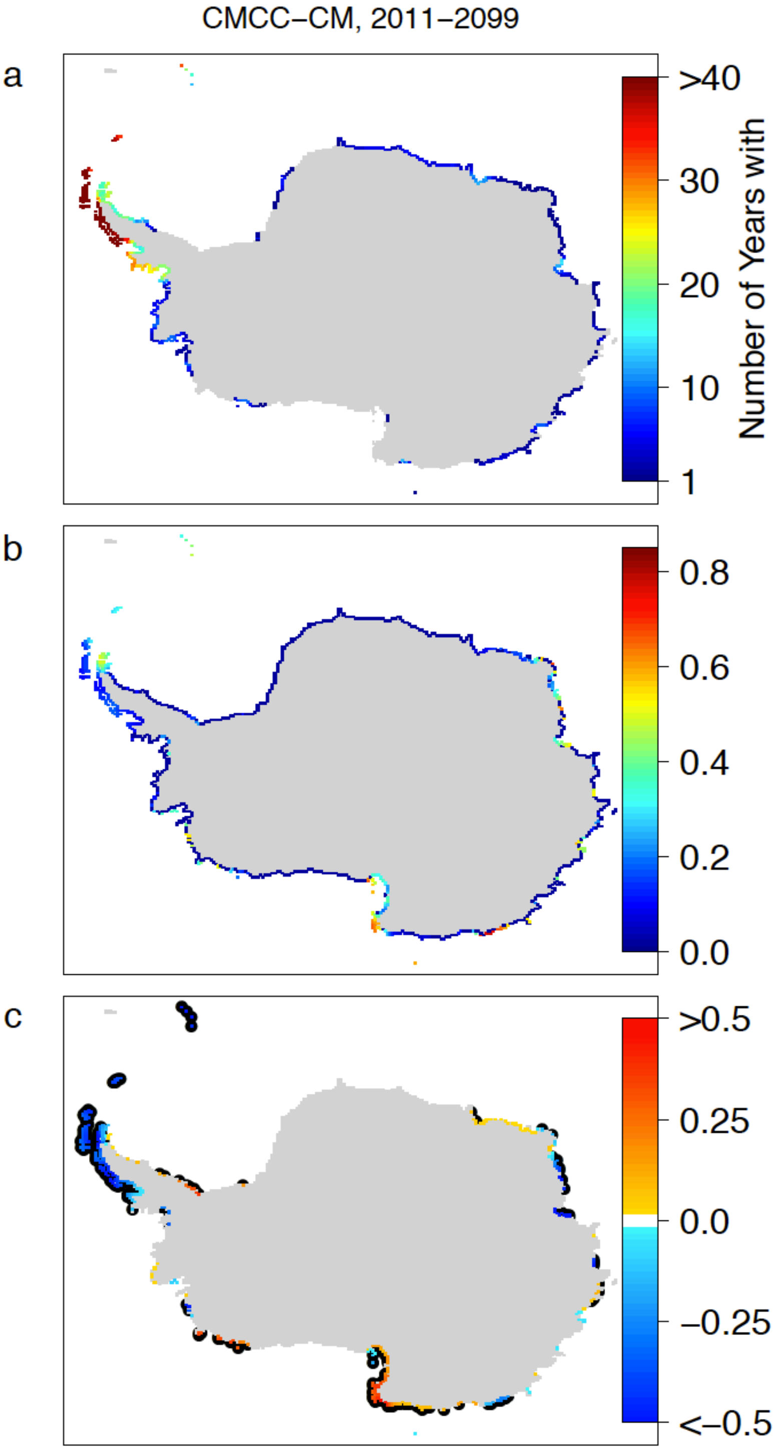

Supplemental Figure 6. Novel climate and Adélie penguin chick-rearing habitat suitability (CRHS) according to CMCC-CM. (**a**) The number of years with novel climate, (**b**) mean CRHS and (**c**) trends in CRHS from General Additive Models using all presence-absence data from 2011-2099. Black outlines represent significant changes over time ($p < 0.05$). The maps were produced in R version 3.1.3 (www.r-project.org).

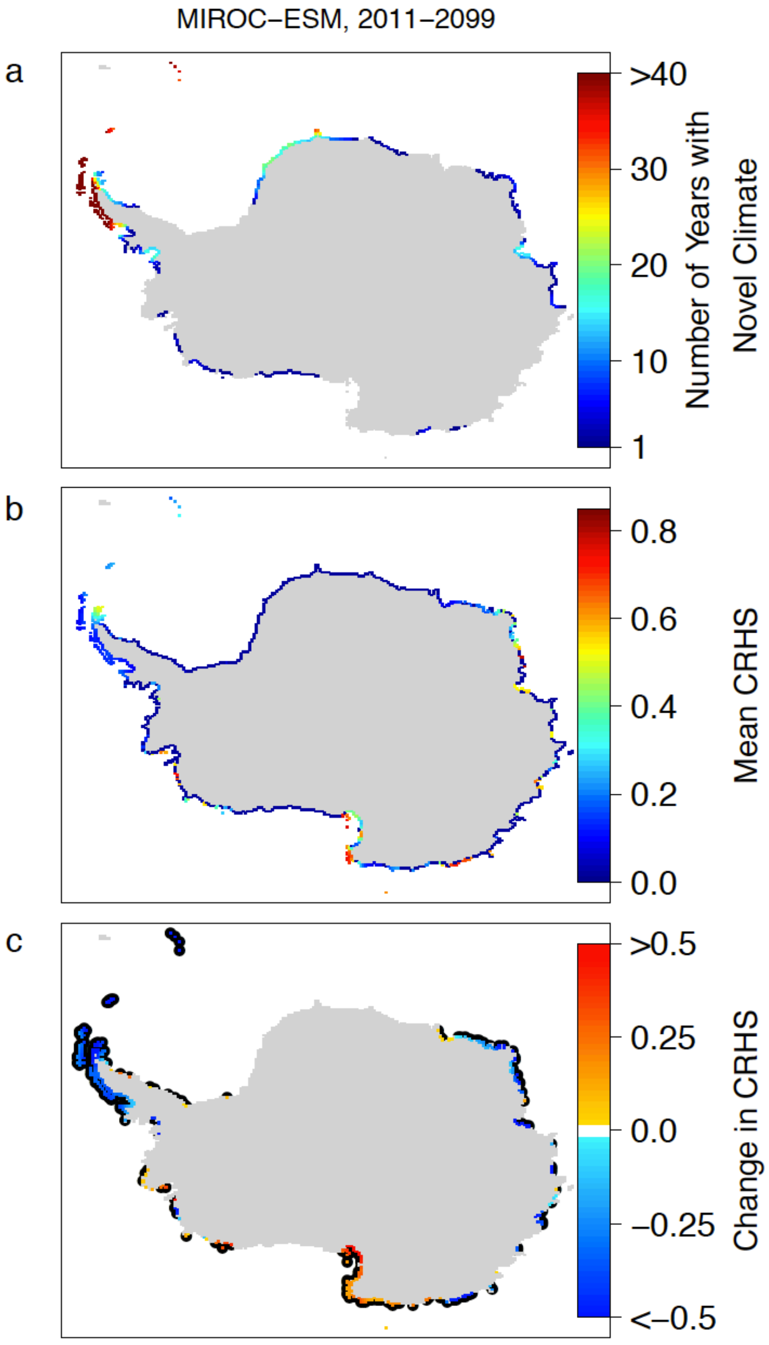

Supplemental Figure 7. Novel climate and Adélie penguin chick-rearing habitat suitability (CRHS) according to MICOC-ESM. (**a**) The number of years with novel climate, (**b**) mean CRHS and (**c**) trends in CRHS from General Additive Models using all presence-absence data from 2011-2099. Black outlines represent significant changes over time ($p < 0.05$). The maps were produced in R version 3.1.3 (www.r-project.org).

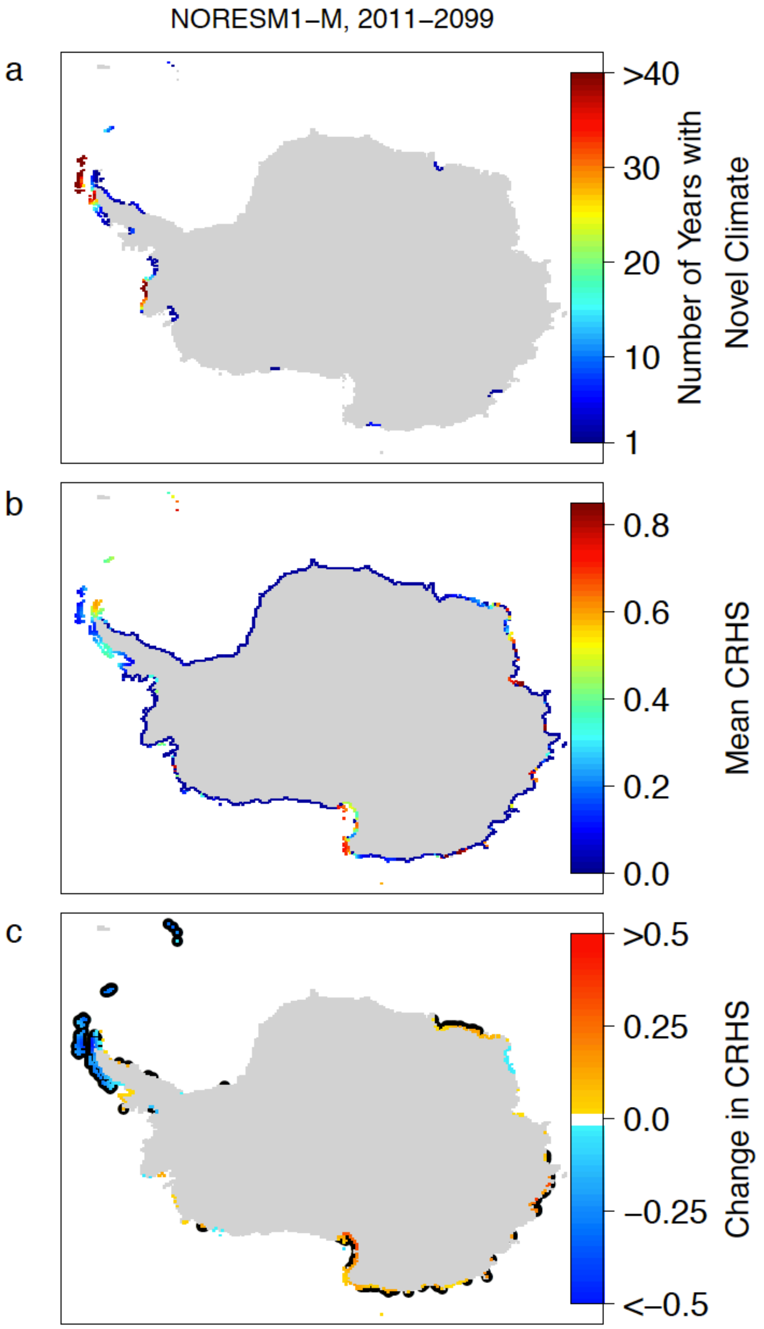

Supplemental Figure 8. Novel climate and Adélie penguin chick-rearing habitat suitability (CRHS) according to NORESM1-M. (**a**) The number of years with novel climate, (**b**) mean CRHS and (**c**) trends in CRHS from General Additive Models using all presence-absence data from 2011-2099. Black outlines represent significant changes over time ($p < 0.05$). The maps were produced in R version 3.1.3 (www.r-project.org).

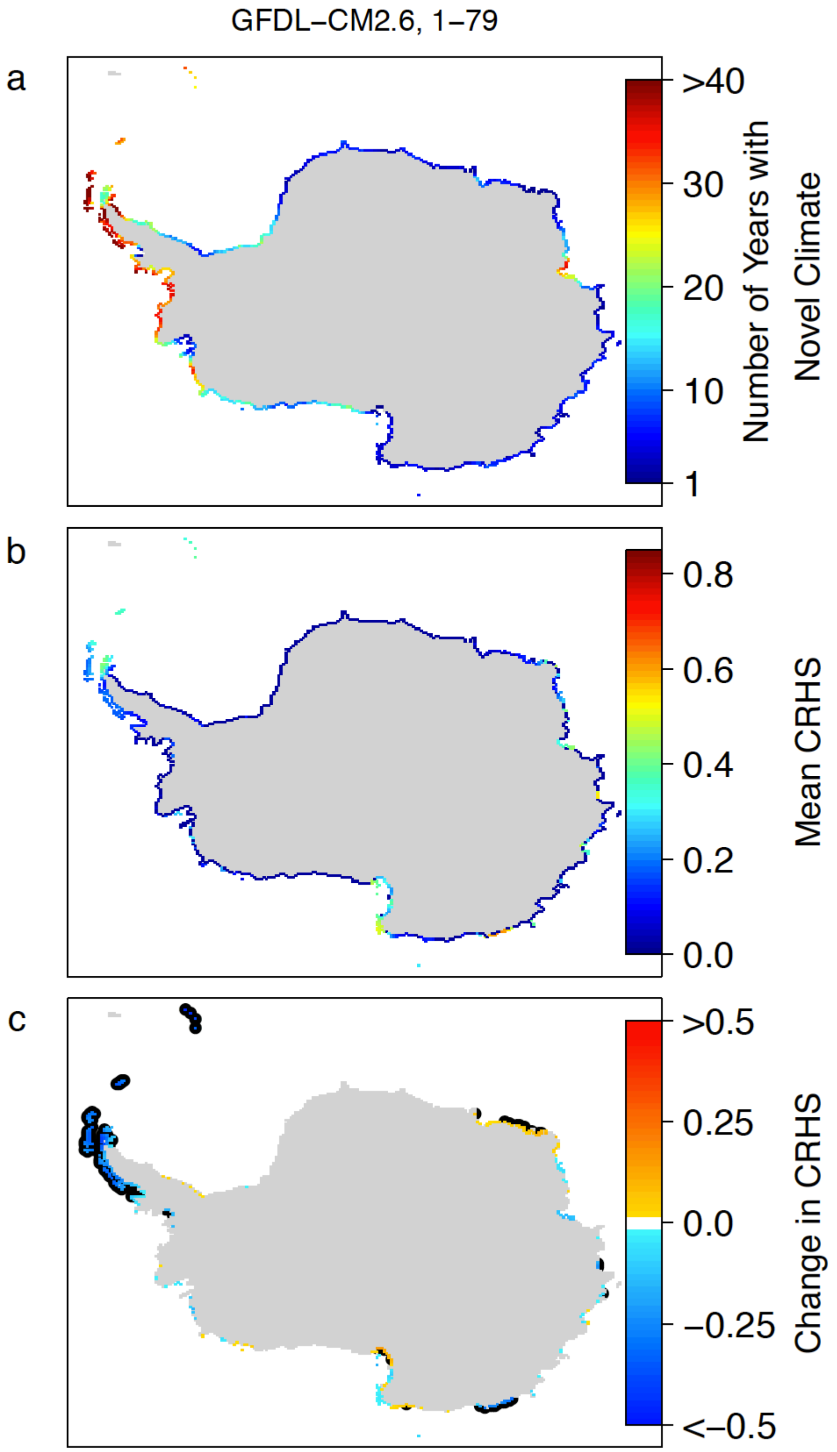

Supplemental Figure 9. Novel climate and Adélie penguin chick-rearing habitat suitability (CRHS) according to GFDL-CM2.6. (**a**) The number of years with novel climate, (**b**) mean CRHS and (**c**) trends in CRHS from General Additive Models using all presence-absence data from model runs 1-79. Black outlines represent significant changes over time ($p < 0.05$). The maps were produced in R version 3.1.3 (www.r-project.org).

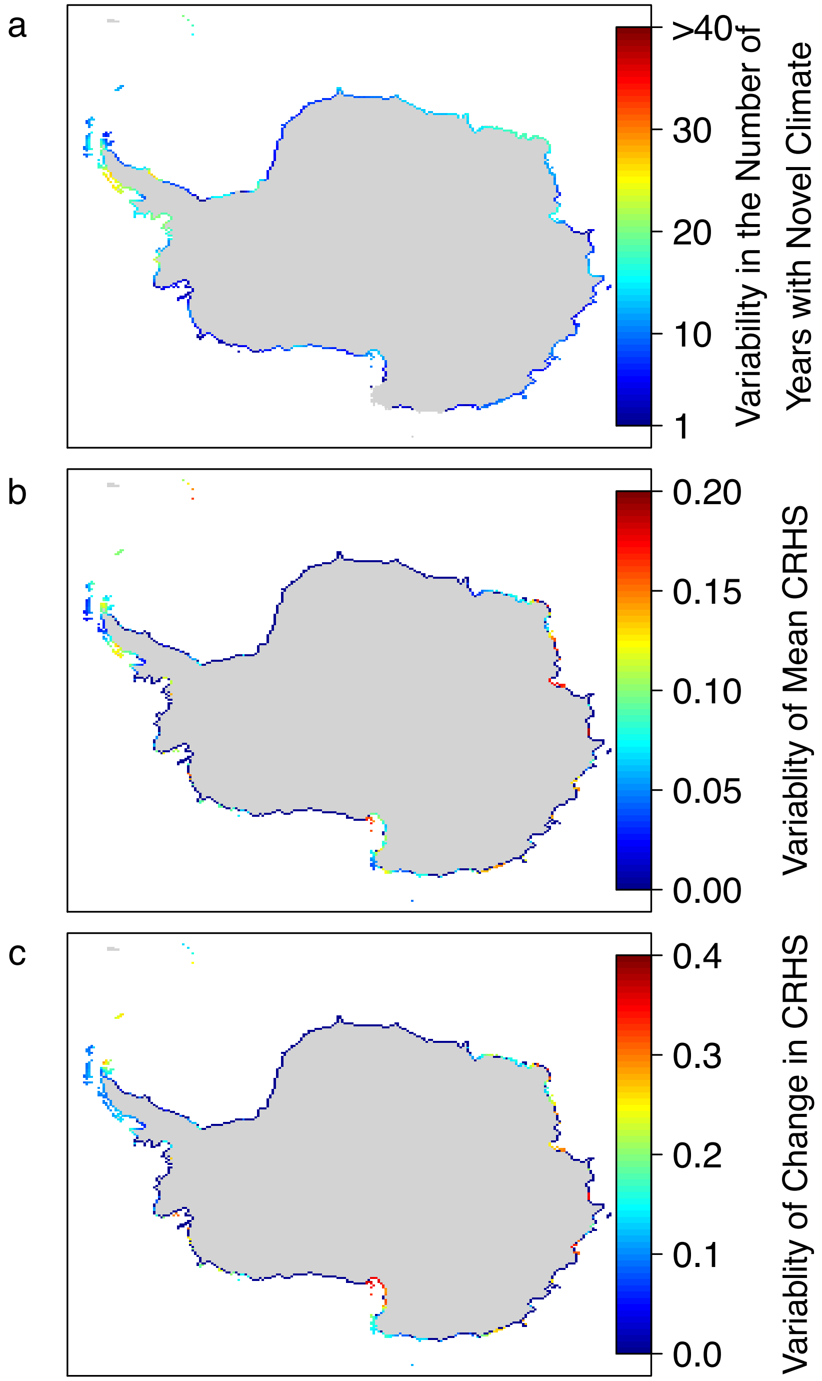

Supplemental Figure 10. Variability in novel climate and Adélie penguin chick-rearing habitat suitability (CRHS) from 2011- 2099. (**a**) The standard deviation of number of years with novel climate, (**b**) standard deviation of CRHS and (**c**) standard deviation in trends in CRHS from an ensemble of global climate models in Fig. 2. The maps were produced in R version 3.1.3 (www.r-project.org).

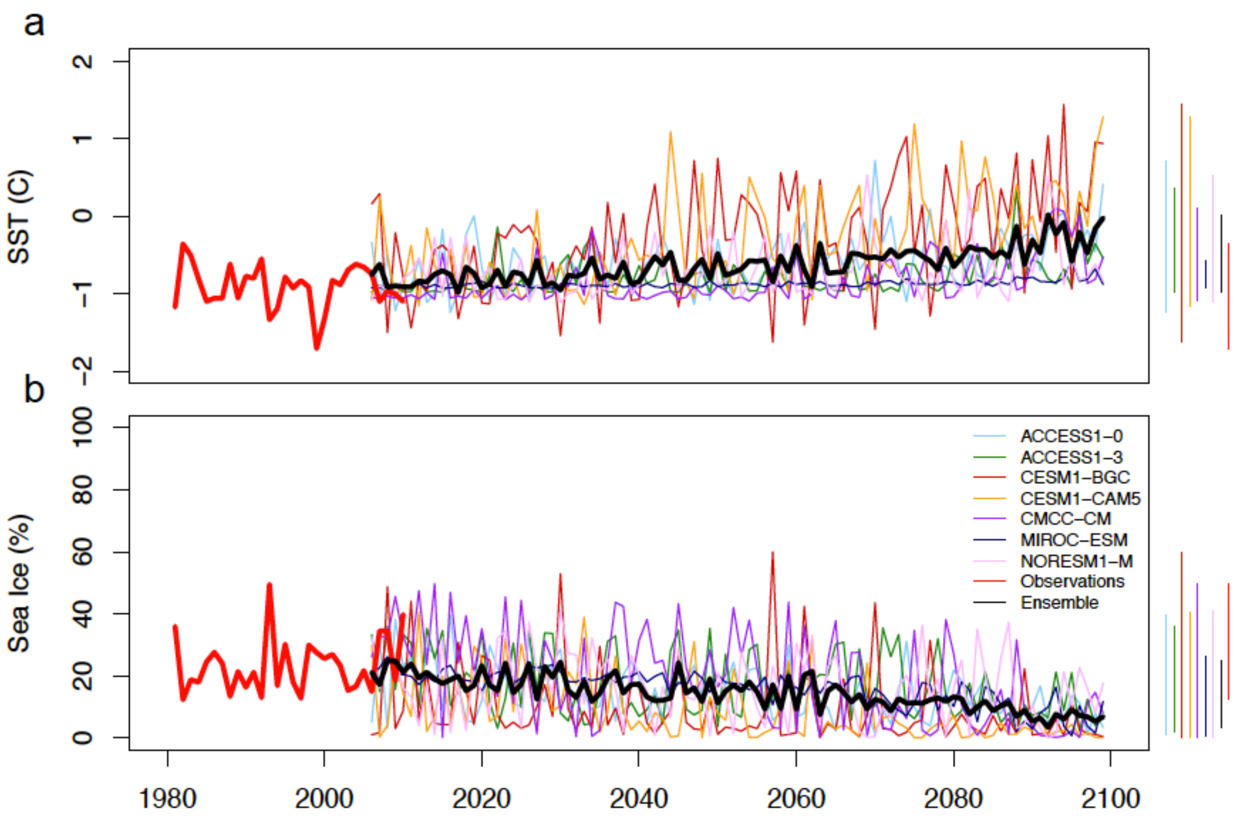

Supplemental Figure 11. (a) Sea surface temperature (SST) and (b) sea ice concentration at Cape Adare, Ross Sea. The satellite observations are in red from 1981-2010, IPCC climate model projections (RCP 8.5) are the colored lines from 2006-2099 and IPCC ensemble is in black. The vertical lines indicate the range of SST and sea ice for satellite data and climate model projections.

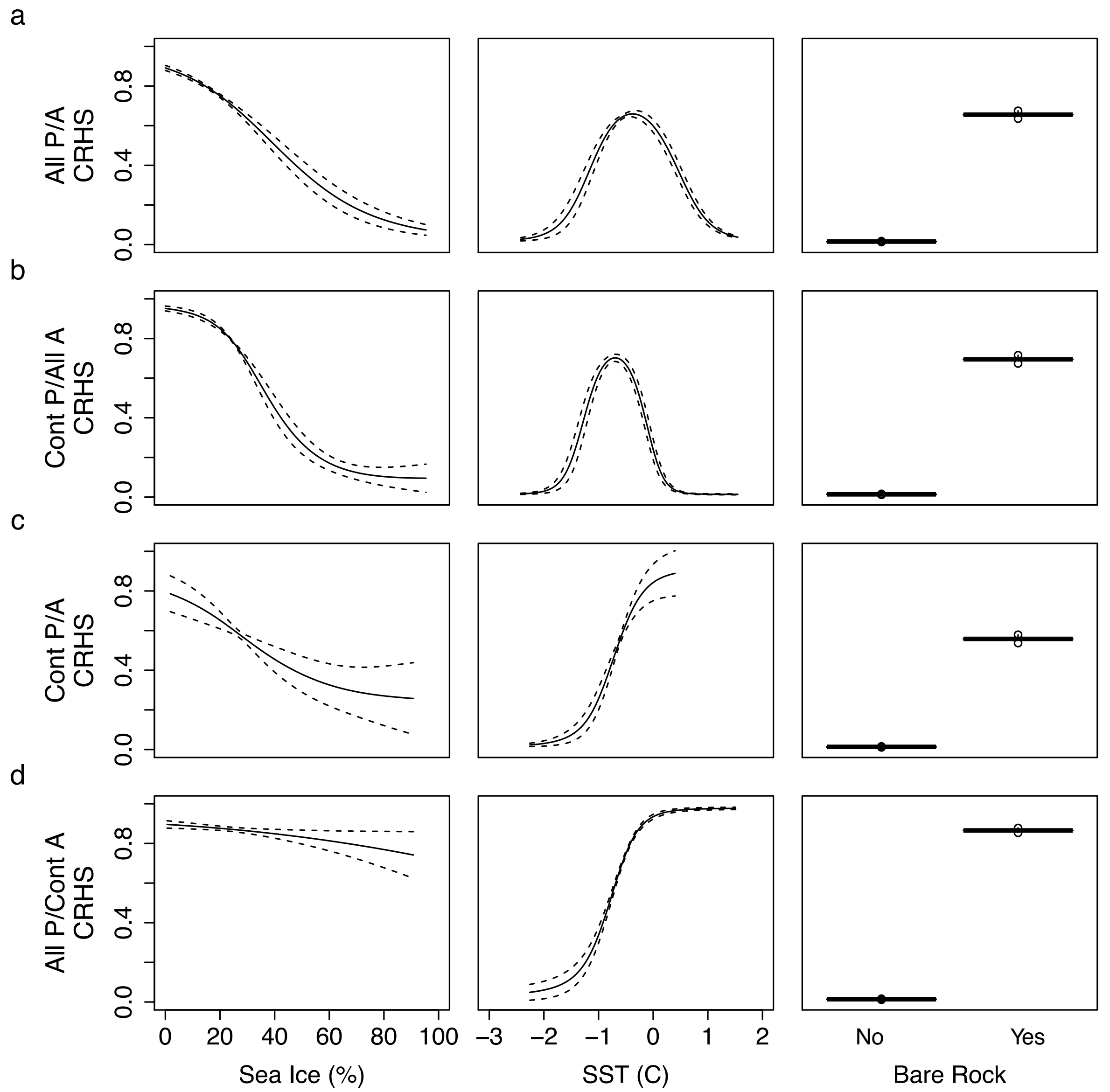

Supplemental Figure 12. **GAM response plot showing the relationship between predicted chick rearing habitat suitability (CRHS) and environmental variables when all other variables were held at their empirical average.** Response plot for sea ice, sea surface temperature (SST) and bare rock for models trained on (**a**) all presence-absence (P/A) data, (**b**) continental (cont) presence and all absence data, (**c**) cont presence/absence data, and (**d**) all presence and cont absence data. The black line is the response curve and the black dashed line is +/- one standard deviation.

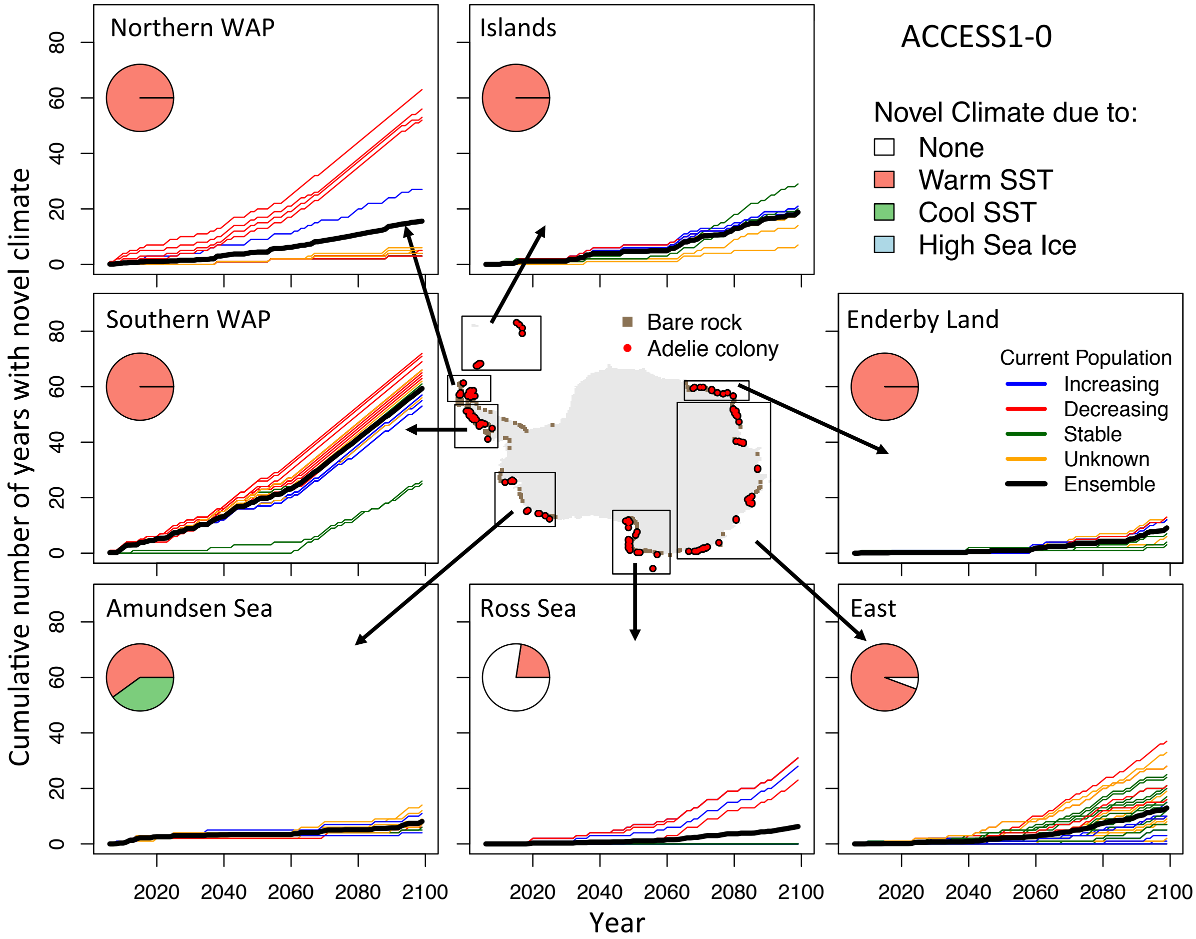

Supplemental Figure 13. The cumulative number of years with novel climate from ACCESS1-0 at Adélie penguin colonies in different Antarctic sectors from 2006-2099. Location map of Adélie penguin colonies and bare rock locations. Each climate model time-series is colored by the current population trend. The ensemble mean shows the average number of years with novel climate for all colonies in a sector. Pie charts show the proportion of colonies in each sector that have no novel climate (none) or the leading cause of novel climate is due to warm sea surface temperature (SST), cool SST or high sea ice concentration. The map was produced in R version 3.1.3 (www.r-project.org).

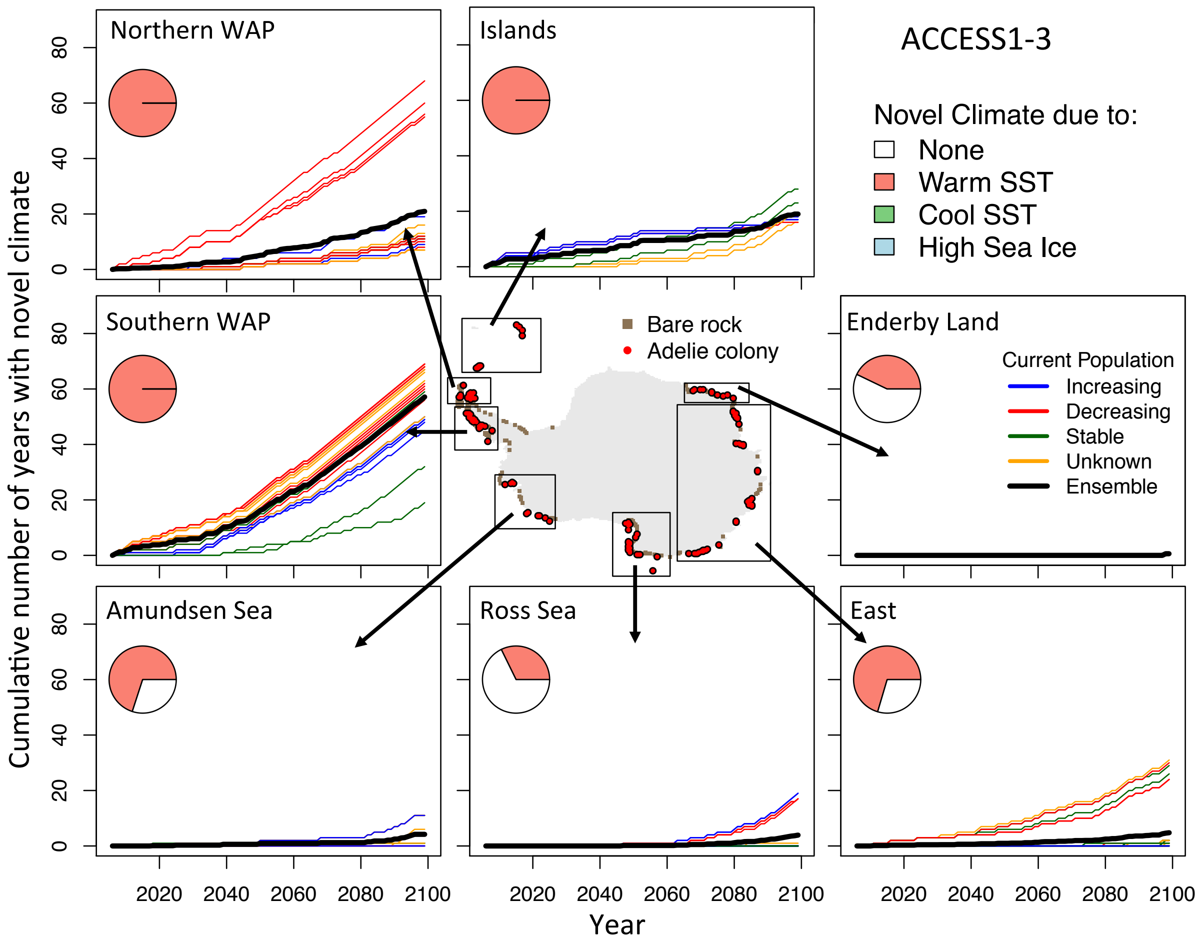

Supplemental Figure 14. **The cumulative number of years with novel climate from ACCESS1-3 at Adélie penguin colonies in different Antarctic sectors from 2006-2099.** Location map of Adélie penguin colonies and bare rock locations. Each climate model time-series is colored by the current population trend. The ensemble mean shows the average number of years with novel climate for all colonies in a sector. Pie charts show the proportion of colonies in each sector that have no novel climate (none) or the leading cause of novel climate is due to warm sea surface temperature (SST), cool SST or high sea ice concentration. The map was produced in R version 3.1.3 (www.r-project.org).

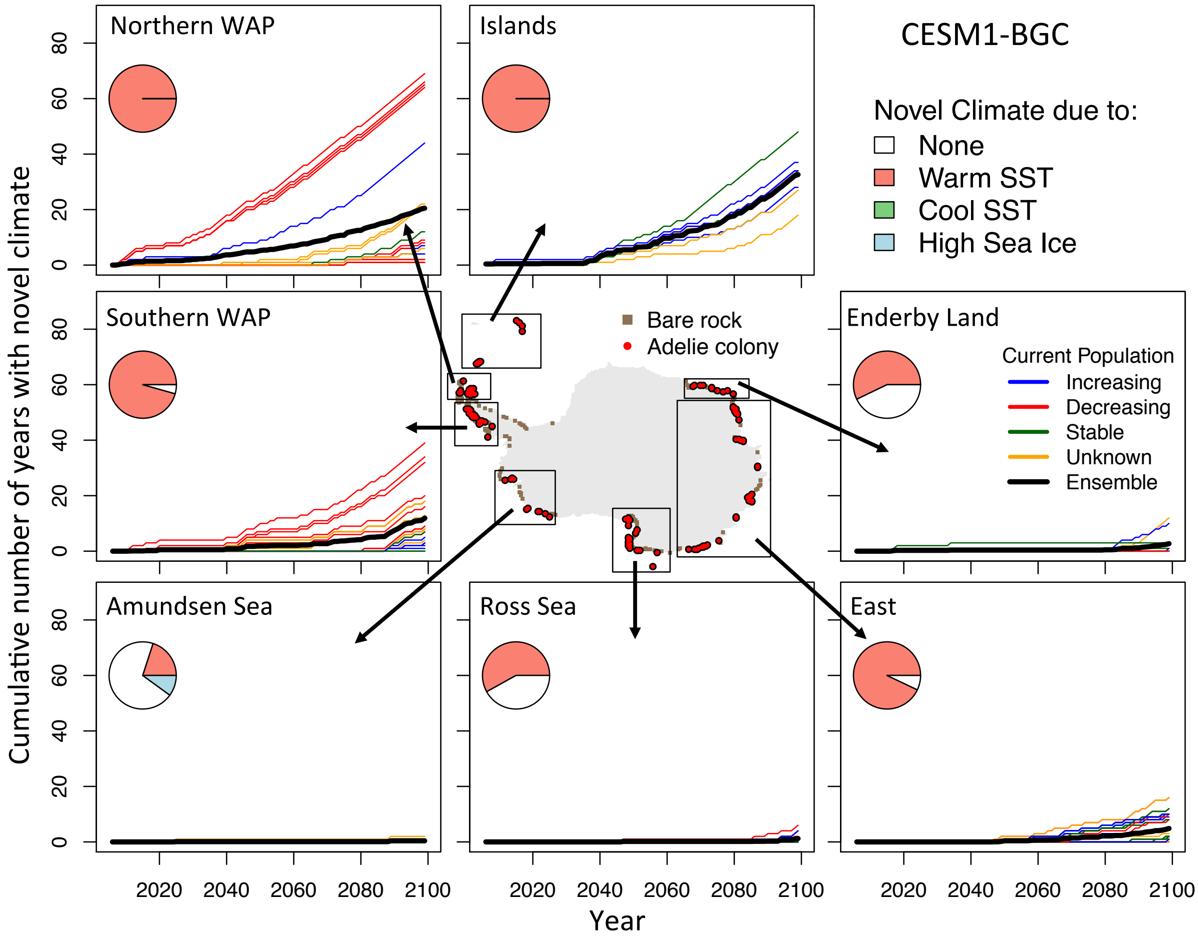

Supplemental Figure 15. **The cumulative number of years with novel climate from CESM1-BGC at Adélie penguin colonies in different Antarctic sectors from 2006-2099.** Location map of Adélie penguin colonies and bare rock locations. Each climate model time-series is colored by the current population trend. The ensemble mean shows the average number of years with novel climate for all colonies in a sector. Pie charts show the proportion of colonies in each sector that have no novel climate (none) or the leading cause of novel climate is due to warm sea surface temperature (SST), cool SST or high sea ice concentration. The map was produced in R version 3.1.3 (www.r-project.org).

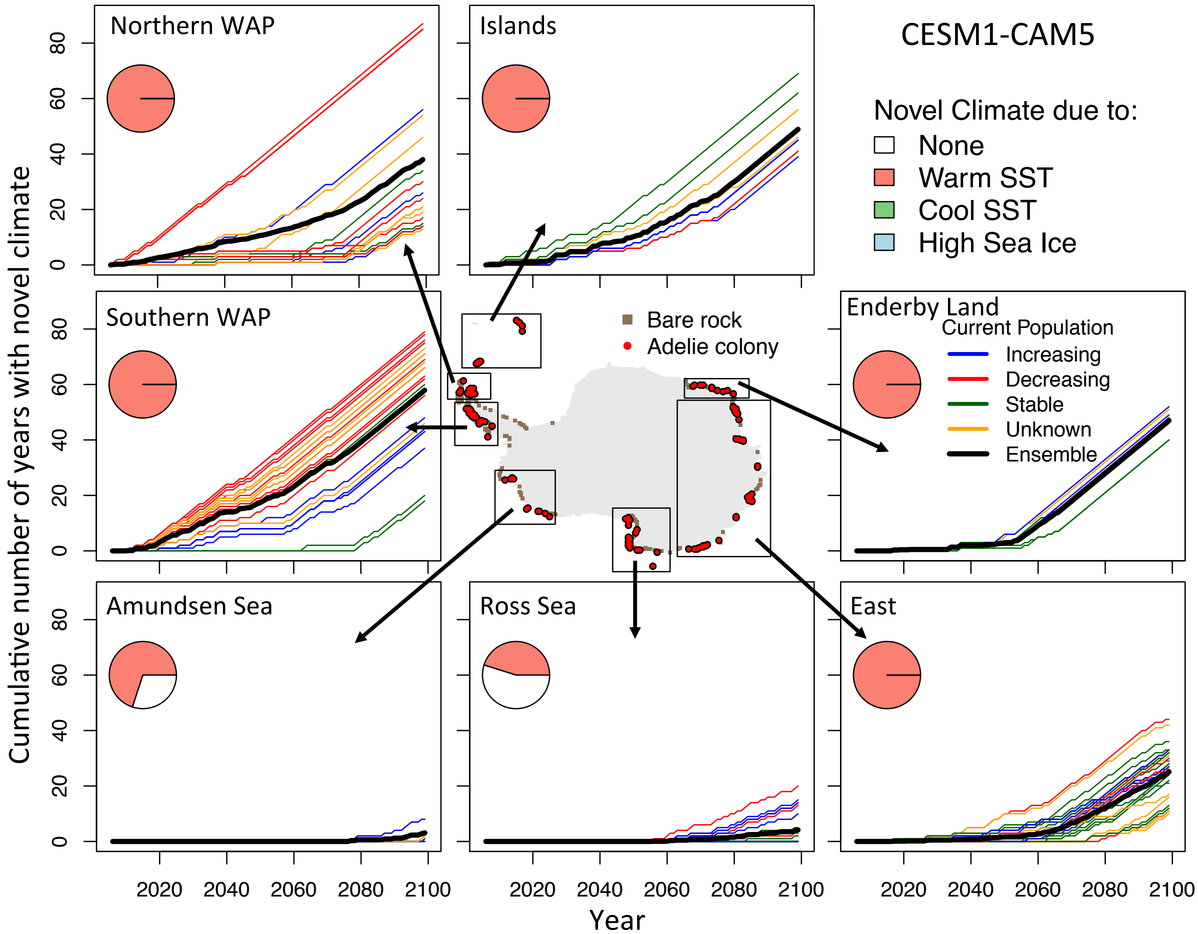

Supplemental Figure 16. **The cumulative number of years with novel climate from CESM1-CAM5 at Adélie penguin colonies in different Antarctic sectors from 2006-2099** Location map of Adélie penguin colonies and bare rock locations. Each climate model time-series is colored by the current population trend. The ensemble mean shows the average number of years with novel climate for all colonies in a sector. Pie charts show the proportion of colonies in each sector that have no novel climate (none) or the leading cause of novel climate is due to warm sea surface temperature (SST), cool SST or high sea ice concentration. The map was produced in R version 3.1.3 (www.r-project.org).

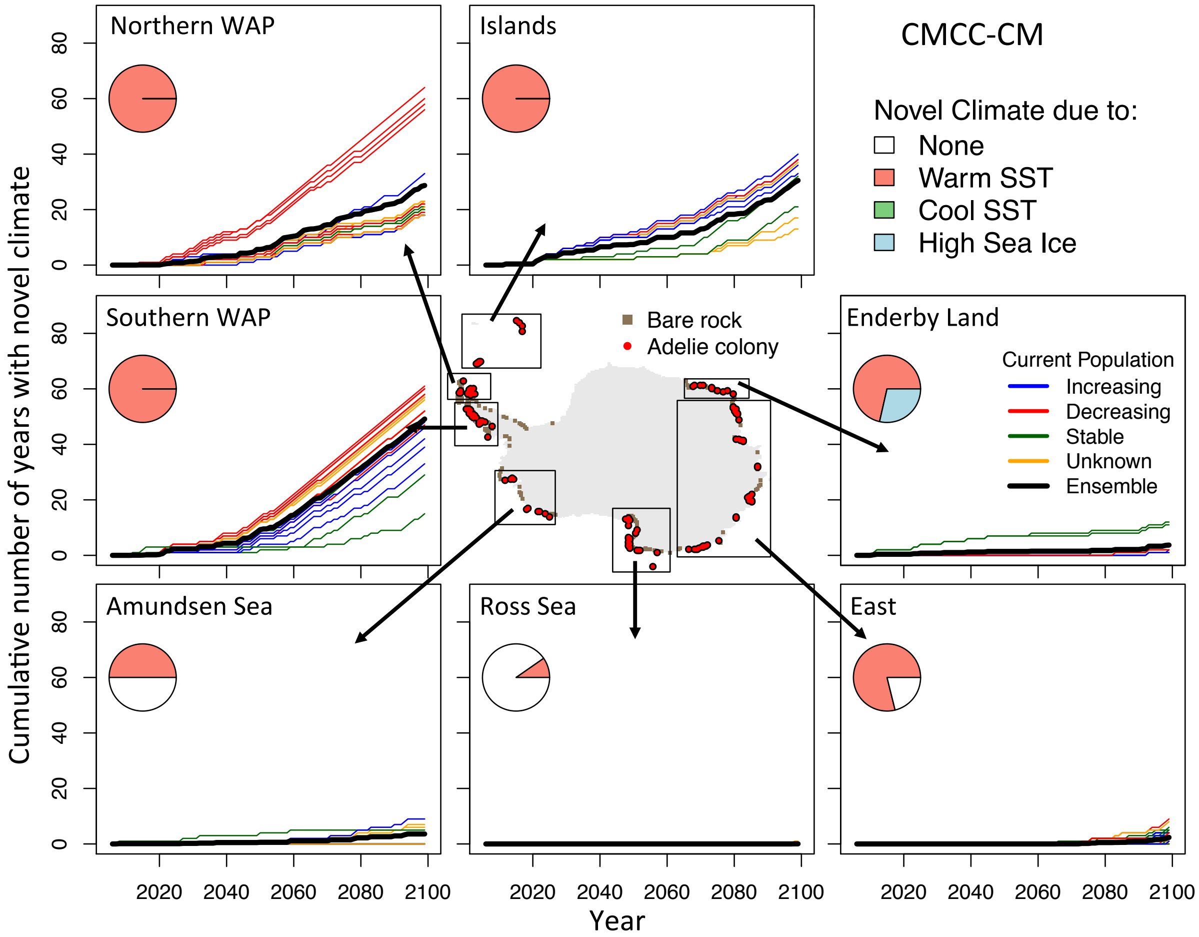

Supplemental Figure 17. **The cumulative number of years with novel climate from CMCC-CM at Adélie penguin colonies in different Antarctic sectors from 2006-2099.** Location map of Adélie penguin colonies and bare rock locations. Each climate model time-series is colored by the current population trend. The ensemble mean shows the average number of years with novel climate for all colonies in a sector. Pie charts show the proportion of colonies in each sector that have no novel climate (none) or the leading cause of novel climate is due to warm sea surface temperature (SST), cool SST or high sea ice concentration. The map was produced in R version 3.1.3 (www.r-project.org).

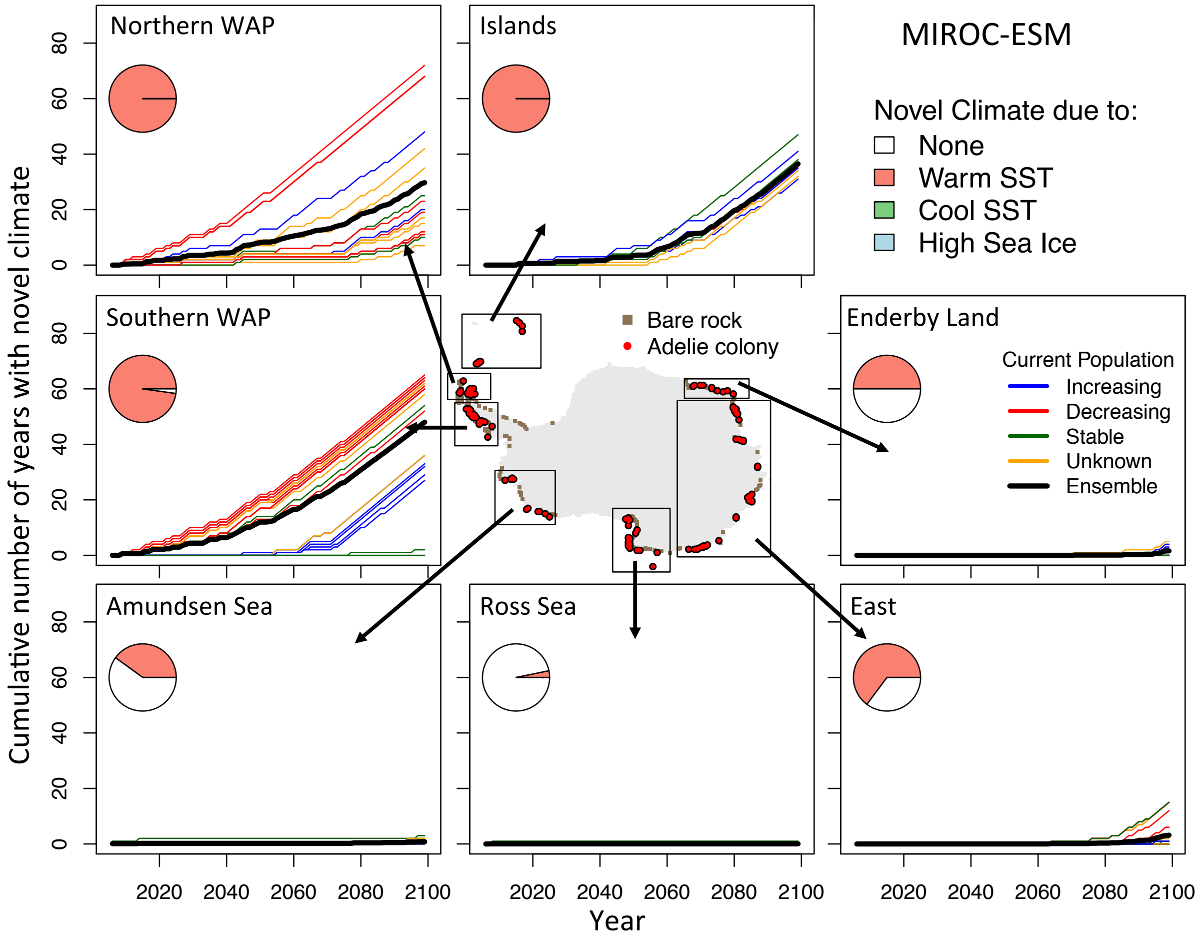

Supplemental Figure 18. **The cumulative number of years with novel climate from MIROC-ESM at Adélie penguin colonies in different Antarctic sectors from 2006-2099.** Location map of Adélie penguin colonies and bare rock locations. Each climate model time-series is colored by the current population trend. The ensemble mean shows the average number of years with novel climate for all colonies in a sector. Pie charts show the proportion of colonies in each sector that have no novel climate (none) or the leading cause of novel climate is due to warm sea surface temperature (SST), cool SST or high sea ice concentration. The map was produced in R version 3.1.3 (www.r-project.org).

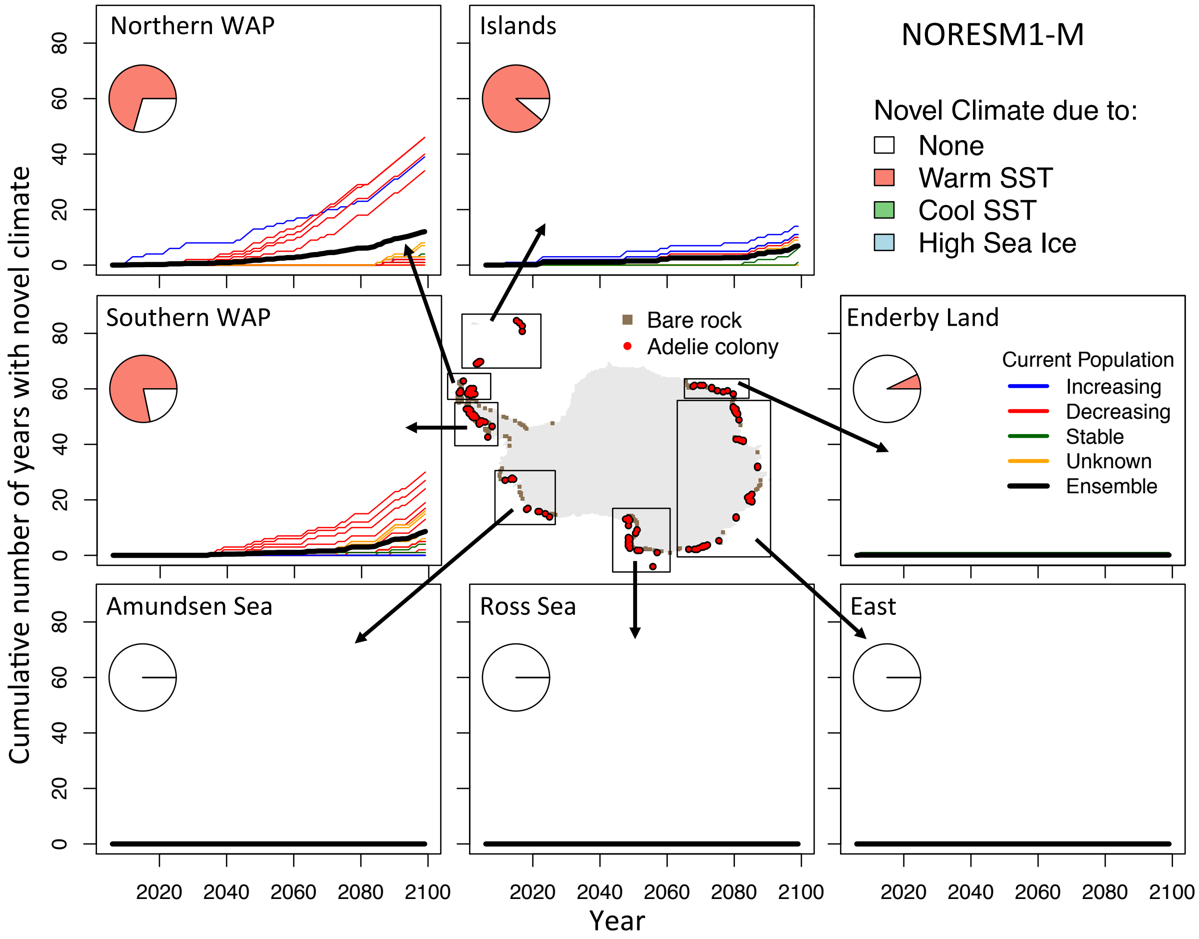

Supplemental Figure 19. **The cumulative number of years with novel climate from NORESM1-M at Adélie penguin colonies in different Antarctic sectors from 2006-2099.** Location map of Adélie penguin colonies and bare rock locations. Each climate model time-series is colored by the current population trend. The ensemble mean shows the average number of years with novel climate for all colonies in a sector. Pie charts show the proportion of colonies in each sector that have no novel climate (none) or the leading cause of novel climate is due to warm sea surface temperature (SST), cool SST or high sea ice concentration. The map was produced in R version 3.1.3 (www.r-project.org).

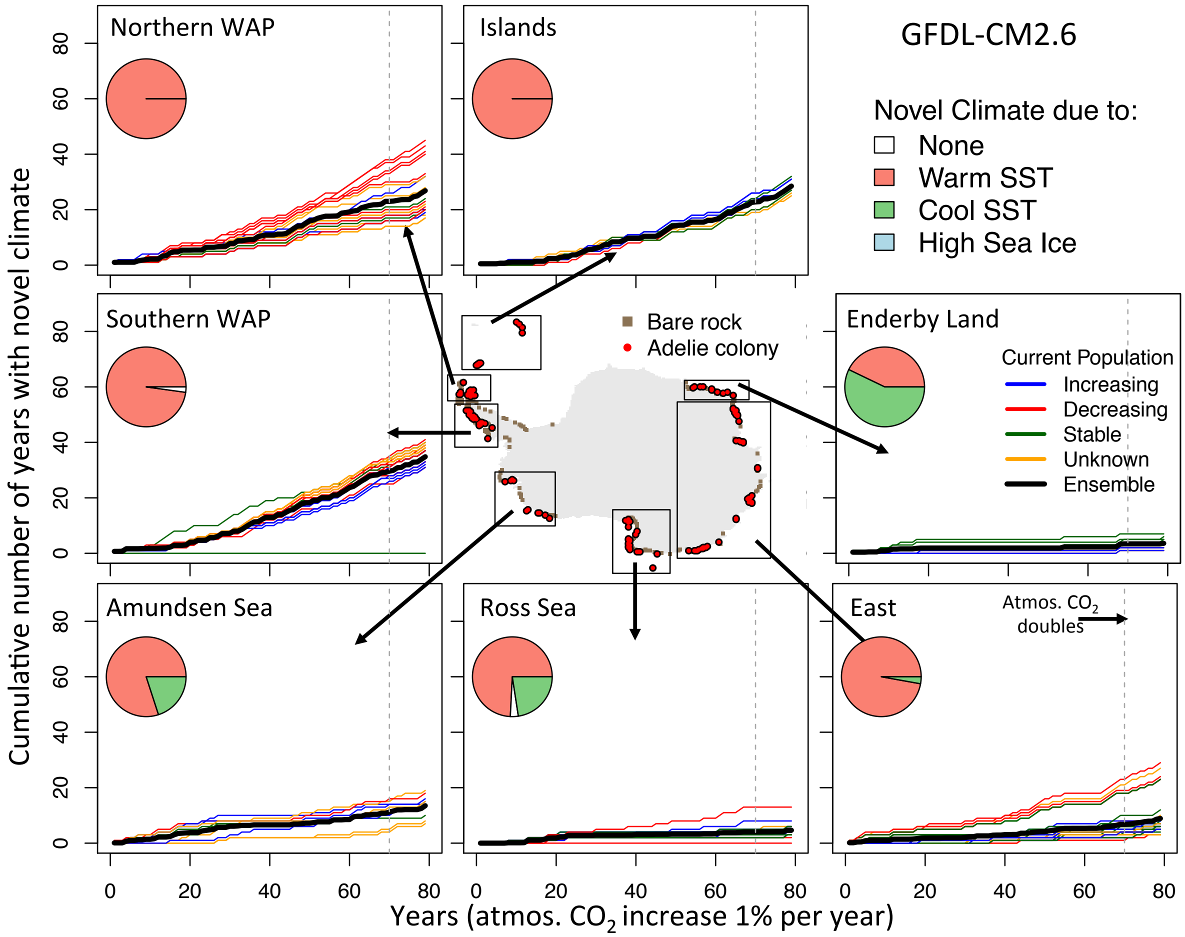

Supplemental Figure 20. **The cumulative number of years with novel climate from GFDL-CM2.6 at Adélie penguin colonies in different Antarctic sectors.** GFDL-CM2.6 ranges from year 1- 79 where atmospheric CO_2 increases by 1% per year and atmospheric CO_2 doubles at year 70 (gray dashed line) in the model simulation. Location map of Adélie penguin colonies and bare rock locations. Each climate model time-series is colored by the current population trend. The ensemble mean shows the average number of years with novel climate for all colonies in a sector. Pie charts show the proportion of colonies in each sector that have no novel climate (none) or the leading cause of novel climate is due to warm sea surface temperature (SST), cool SST or high sea ice concentration. The map was produced in R version 3.1.3 (www.r-project.org).

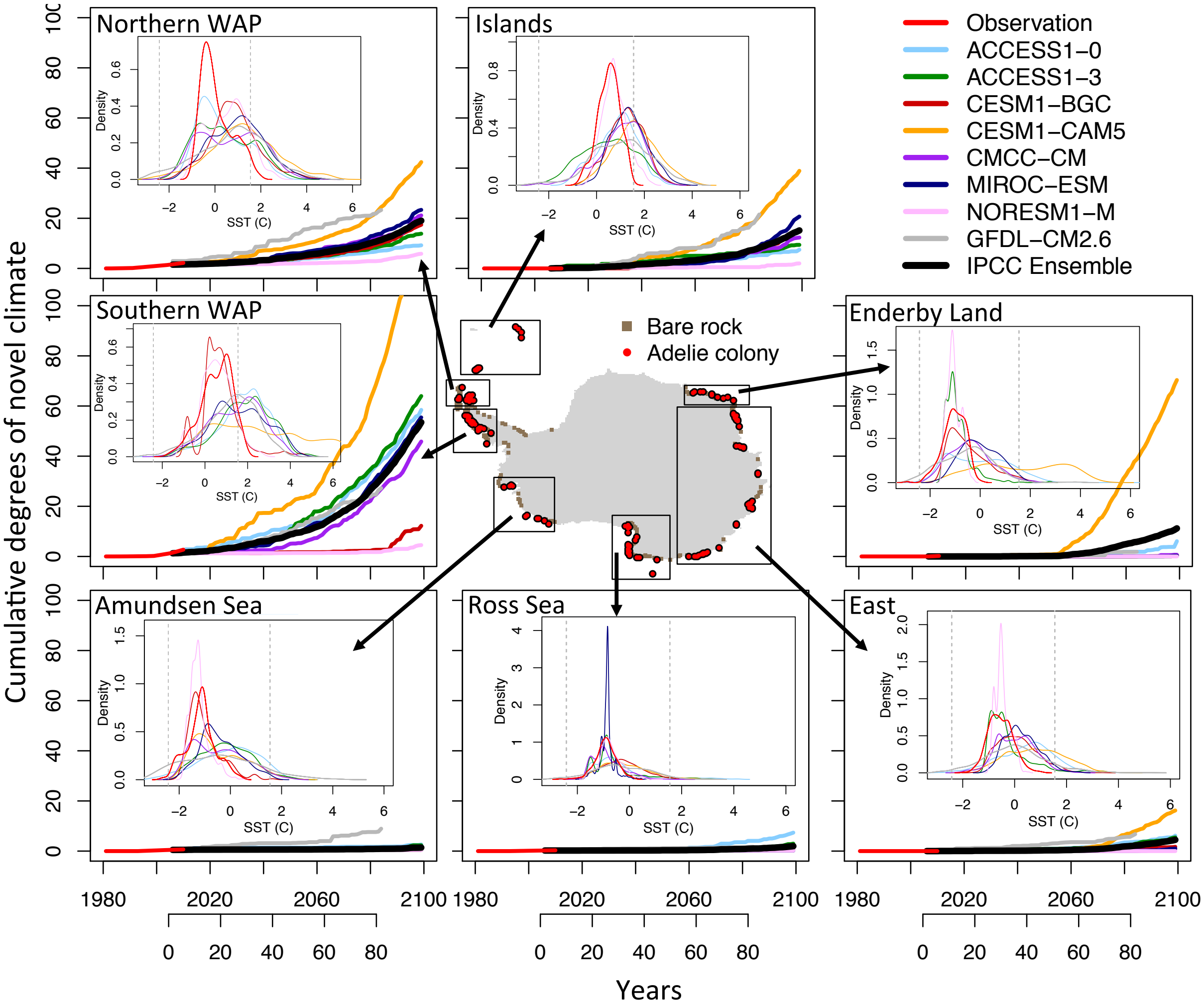

Supplemental Figure 21. The cumulative degrees of novel climate and distribution of SST at Adélie penguin colonies in different Antarctic sectors. To calculate cumulative degrees the absolute value of the difference of SST from novel thresholds was taken (-2.42ºC was subtracted from negative values and 1.55ºC was subtracted from positive values). Each line represents the mean of all colonies in that sector colored by the climate model used for the projection. Satellite observations of novel climate are in red from 1981-2010, IPCC models (RCP 8.5) from 2006-2099 and GFDL-CM2.6 from year 1-79 where atmospheric CO_2 increases by 1% per year and atmospheric CO_2 doubles at year 70 in the model simulation. The IPCC ensemble mean shows the average trend for all IPCC models in a sector (not including GFDL-CM2.6). The subplot shows the kernel density estimate of SST within each sector for each climate model and satellite observations. The vertical dashed lines represent the novel climate thresholds. The location map of Adélie penguin colonies and bare rock locations was produced in R version 3.1.3 (www.r-project.org).

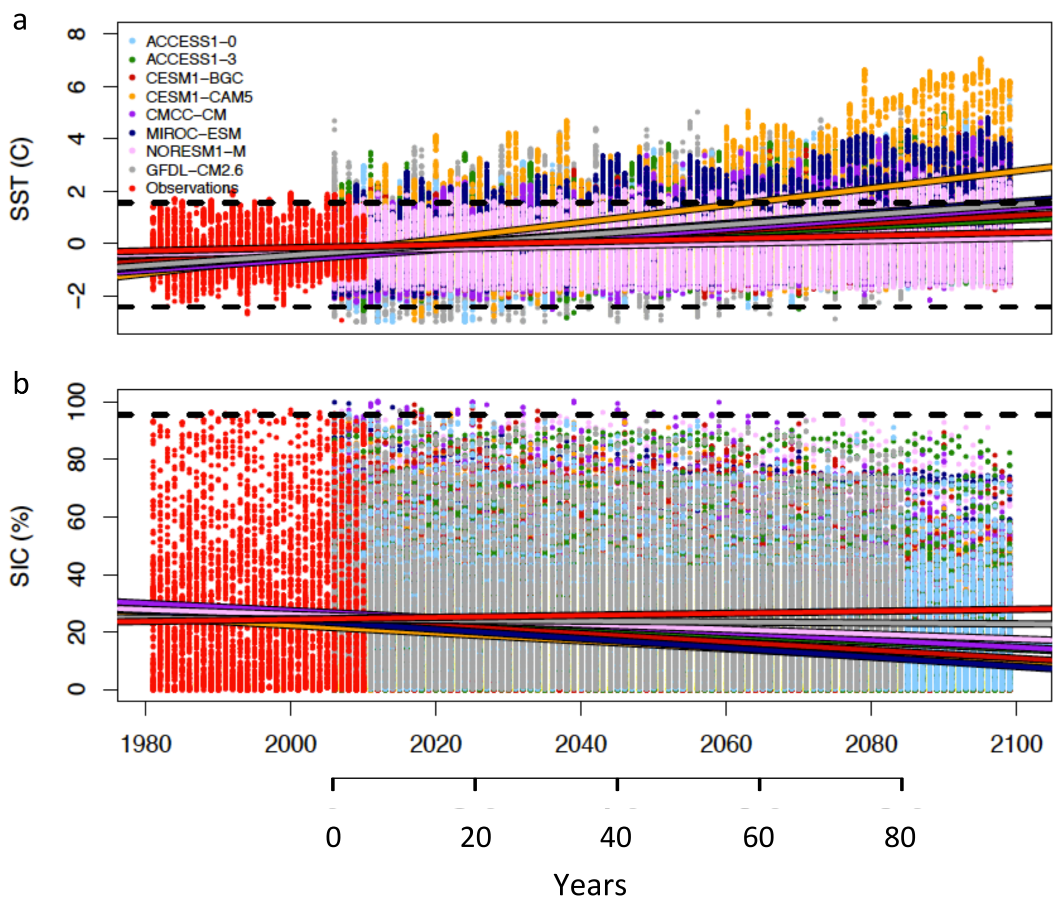

Supplemental Figure 22. Satellite observations and global climate model projections around the Antarctic coastline from 1981-2099 for a) sea surface temperature (SST) and b) sea ice concentration (SIC). Colored lines represent the linear regression of the mean condition for each year for each dataset. Novel conditions (-2.42 > SST > 1.55ºC, SIC > 95.41%) are above or below black horizontal dashed lines. Satellite observations are from 1981-2010, IPCC models from 2006-2099 and GFDL-CM2.6 from year 1-79.

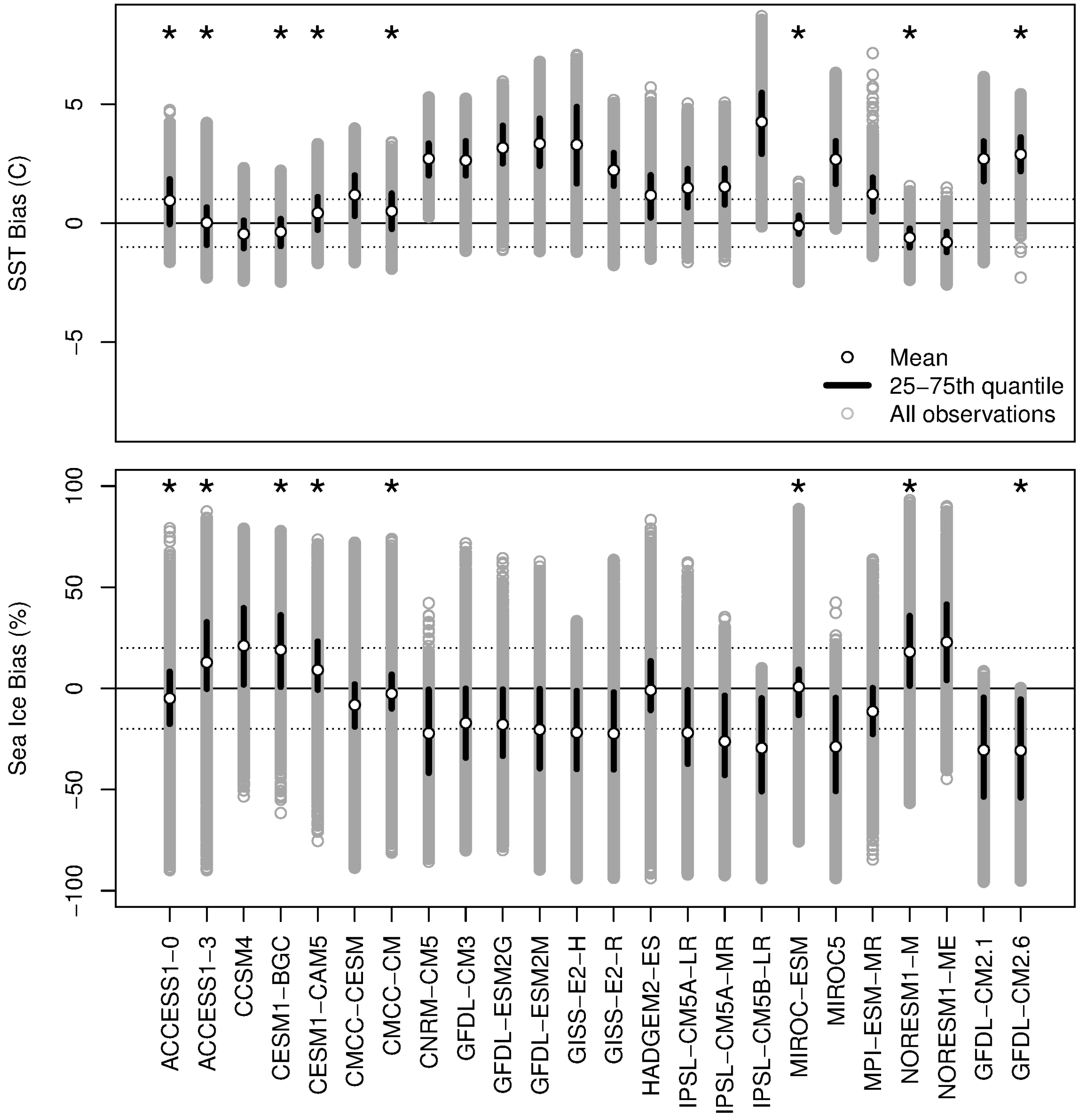

Supplemental Figure 23. Comparison of CMIP5 and two additional NOAA GFDL models showing austral summer (DJF) sea ice and sea surface temperature (SST) mean bias within 200 km of land around Antarctica. (**a**) Mean SST bias (model minus observations) and (**b**) Mean sea ice concentration bias relative to mean observations from the satellite record **(**1981-2004 SST, 1978-2004 Sea Ice**)**. Mean model output from the CMIP5 models is from the IPCC AR5 historical runs (years 1978-2004). Mean model output from NOAA GFDL's CM2.1 and CM2.6 is from years 101-140 of the 1990 control simulation (global atmospheric CO_2 fixed at the 1990 level). The * symbol indicates the models chosen for further analysis (mean bias within ±1C and ±20% sea ice, represented by horizontal dashed lines, and GFDL-CM2.6 was chosen because it is a high-resolution model).

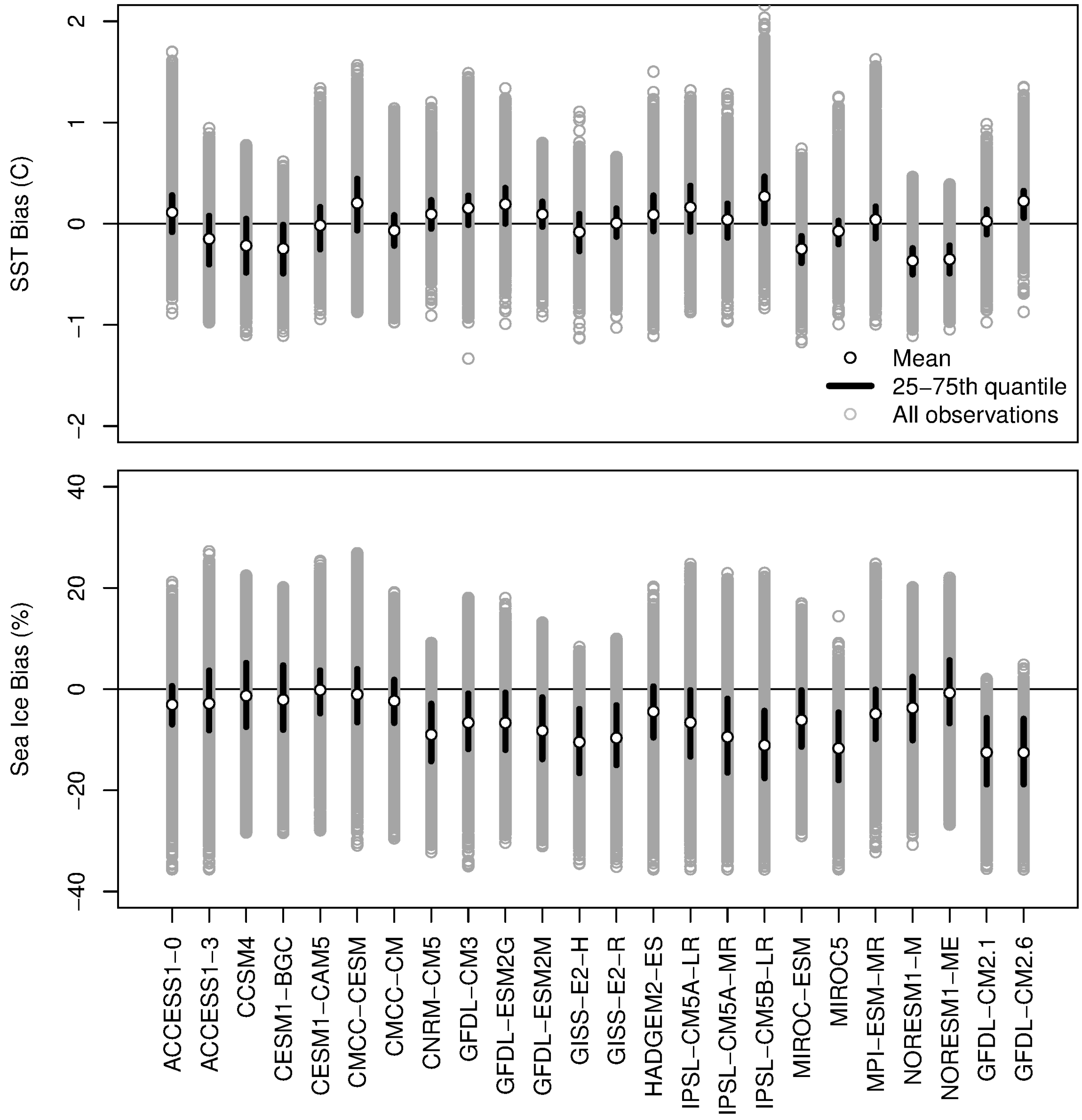

Supplemental Figure 24. Comparison of CMIP5 and two additional NOAA GFDL models showing austral summer (DJF) sea ice and sea surface temperature (SST) standard deviation bias within 200 km of land around Antarctica. (**a**) Standard deviation of SST bias (model minus observations) and (**b**) Standard deviation of sea ice concentration bias relative to mean observations from the satellite record **(**1981-2004 SST, 1978-2004 Sea Ice**)**. Mean model output from the CMIP5 models is from the IPCC AR5 historical runs (years 1978-2004). Standard deviation of model output from NOAA GFDL's CM2.1 and CM2.6 is from years 101-140 of the 1990 control simulation (global atmospheric CO_2 fixed at the 1990 level). The * symbol indicates the models chosen for further analysis.

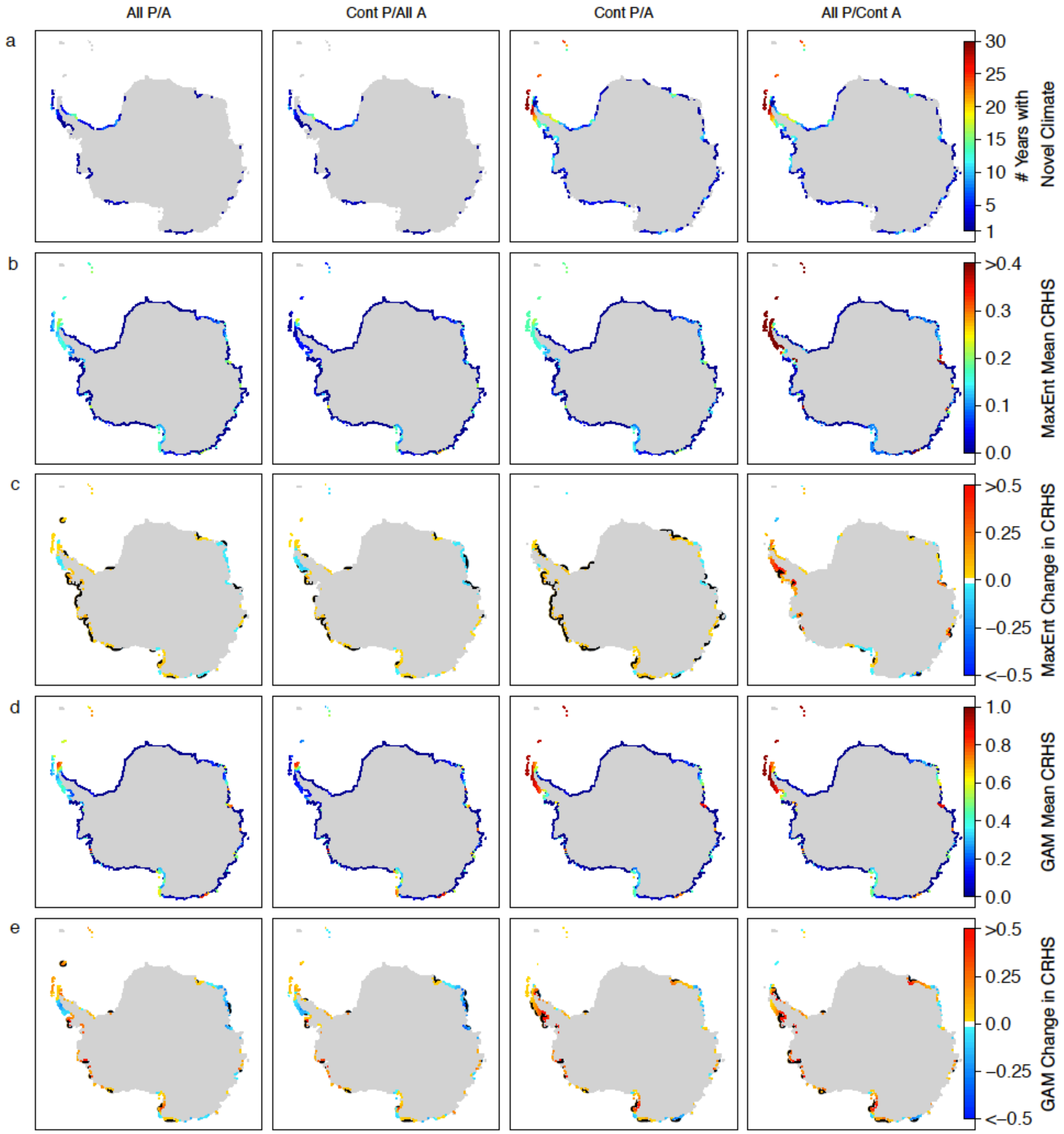

Supplemental Figure 25. Comparison of chick-rearing habitat suitability (CRHS) from MaxEnt and Generalized Additive Models (GAM) using different combinations of presence-absence (P/A) data from 1981-2010. Each column specifies which combination of P/A data was used to train the model (Cont = continental). **(a)** The number of years a pixel had novel climate data, which is data outside the range of the training data. (**b**) Mean and (**c**) change in CRHS from MaxEnt models. (**d**) Mean and (**e**) change in CRHS from GAMs. Black shading represent significant trends over time ($p < 0.05$). The maps were produced in R version 3.1.3 (www.r-project.org).

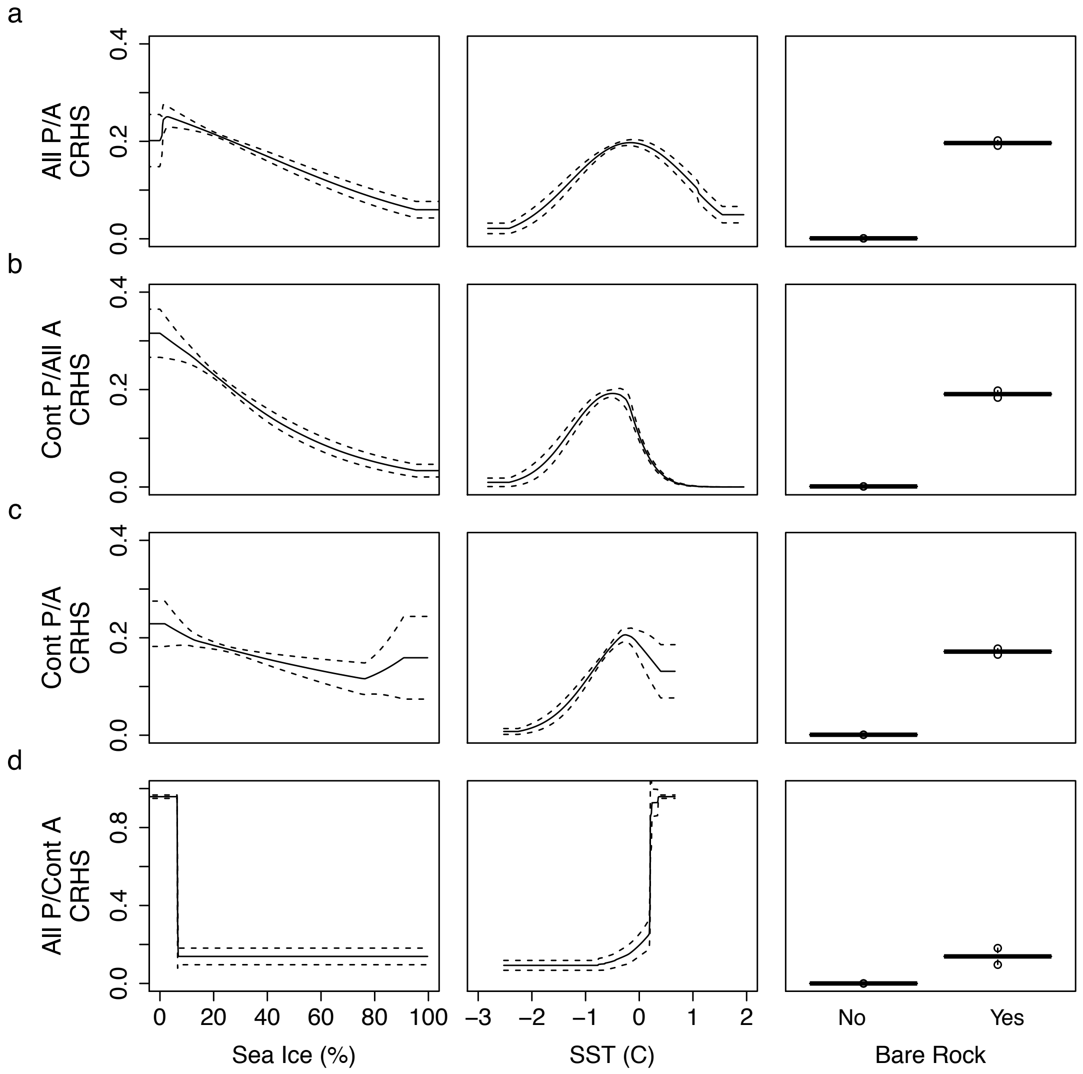

Supplemental Figure 26. **MaxEnt response plot showing the relationship between predicted chick rearing habitat suitability (CRHS) and environmental variables when all other variables were held at their empirical average.** Response plot for sea ice, sea surface temperature (SST) and bare rock for models trained on (**a**) all presence-absence (P/A) data, (**b**) continental (cont) presence and all absence data, (**c**) cont presence/absence data, and (**d**) all presence and cont absence data. The black line is the response curve and the black dashed line is +/- one standard deviation.

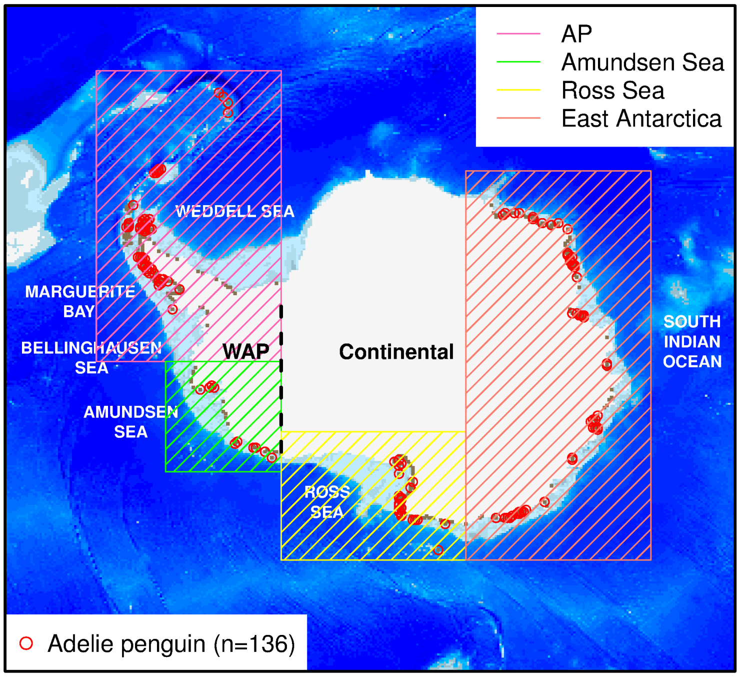

Supplemental Figure 27. Adélie penguin breeding colonies in the Southern Ocean. The black dashed line separates West Antarctic Peninsula (WAP) from continental Adélie penguin colonies. Bare rock (■) locations along the coastline and light to dark blue represents shallow to deep bathymetry. Penguin colonies were grouped into four Antarctic sectors according to the boxes. AP = Antarctic Peninsula. The map was produced in R version 3.1.3 (www.r-project.org).

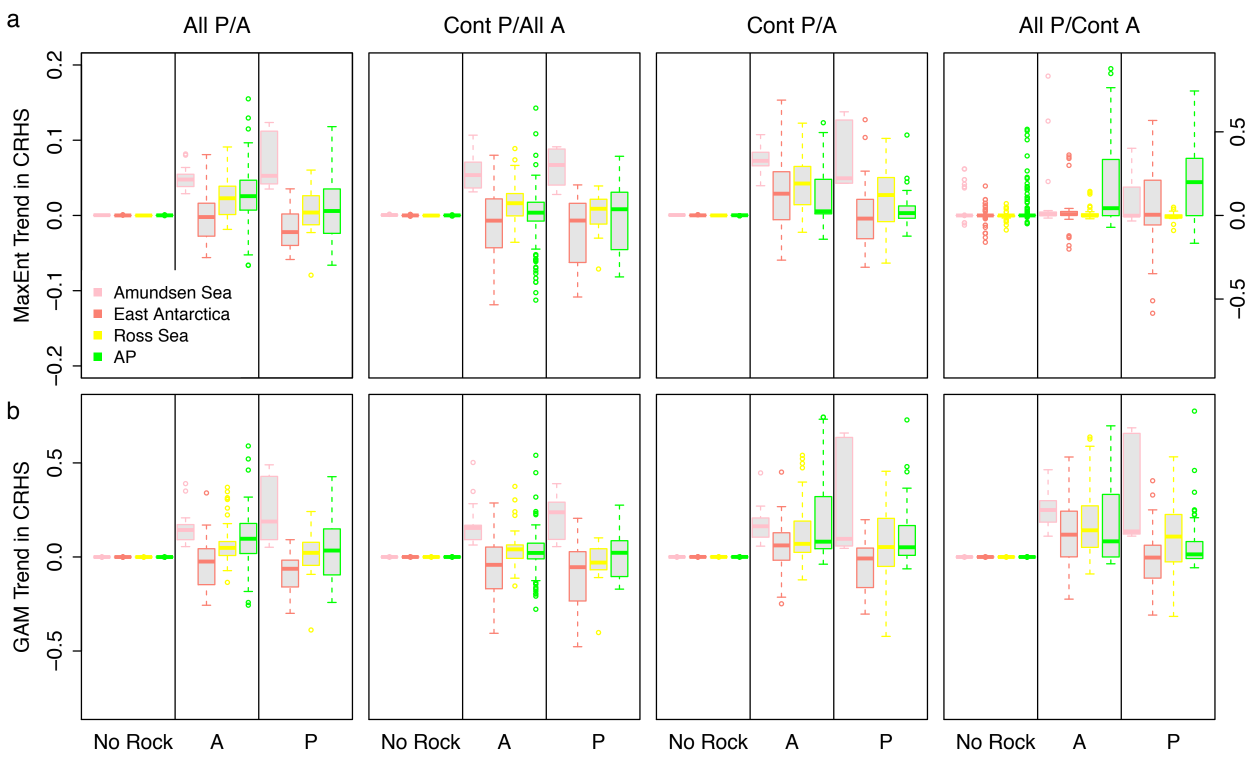

Supplemental Figure 28. **Comparison of trends in chick rearing habitat suitability (CRHS) in four different Antarctic sectors (see map in Supplemental Fig. 27).** Trends in CRHS at locations with no bare rock, absent (A) locations, and present (P) locations according to (**a**) MaxEnt models and (**b**) Generalized Additive Models (GAMs) trained with different combinations of presence-absence (P/A) data (cont = continental). Note the scale for Maxent trends for All P/Cont A model is different than the other Maxent trends.

Supplemental Tables

Supplemental Table 1. Climate models and groups from the Intergovernmental Panel on Climate Change (IPCC) assessment report (AR5) and two additional NOAA GFDL models that we used in our analyses.

**Model**	**Modeling group**
ACCESS1-0	CSIRO (Commonwealth Scientific and Industrial Research Organisation, Australia), and BOM (Bureau of Meteorology, Australia)
ACCESS1-3	CSIRO (Commonwealth Scientific and Industrial Research Organisation, Australia), and BOM (Bureau of Meteorology, Australia)
CCSM4	National Center for Atmospheric Research
CESM1-BGC	National Science Foundation, Department of Energy, National Center for Atmospheric Research
CESM1-CAM5	National Science Foundation, Department of Energy, National Center for Atmospheric Research
CMCC-CESM	Centro Euro-Mediterraneo per I Cambiamenti Climatici
CMCC-CM	Centro Euro-Mediterraneo per I Cambiamenti Climatici
CNRM-CM5	Centre National de Recherches Meteorologiques / Centre Europeen de Recherche et Formation Avancees en Calcul Scientifique
GFDL-CM3	NOAA Geophysical Fluid Dynamics Laboratory
GFDL-ESM2G	NOAA Geophysical Fluid Dynamics Laboratory
GFDL-ESM2M	NOAA Geophysical Fluid Dynamics Laboratory
GISS-E2-H	NASA Goddard Institute for Space Studies
GISS-E2-R	NASA Goddard Institute for Space Studies
HADGEM2-ES	National Institute of Meteorological Research/Korea Meteorological Administration
IPSL-CM5A-LR	Institut Pierre-Simon Laplace
IPSL-CM5A-MR	Institut Pierre-Simon Laplace
IPSL-CM5B-LR	Institut Pierre-Simon Laplace
MIROC-ESM	Japan Agency for Marine-Earth Science and Technology, Atmosphere and Ocean Research Institute (The University of Tokyo), and National Institute for Environmental Studies
MIROC5	Atmosphere and Ocean Research Institute (The University of Tokyo), National Institute for Environmental Studies, and Japan Agency for Marine-Earth Science and Technology
MPI-ESM-MR	Max Planck Institute for Meteorology (MPI-M)
NORESM1-M	Norwegian Climate Centre
NORESM1-ME	Norwegian Climate Centre
GFDL-CM2.1	NOAA Geophysical Fluid Dynamics Laboratory
GFDL-CM2.6	NOAA Geophysical Fluid Dynamics Laboratory

Supplemental Table 2. Variable importance (VI, %) and area under the curve (AUC) for Generalized Additive Models (GAMs) and MaxEnt models (mean ± standard deviation) with different combinations of presence-absence (P/A) data (Cont = continental).

		All P/A Model	Cont. P/ All A Model	Cont. P/A Model	All P/ Cont A Model
GAM	VI Bare Rock	54.69 ± 1.97	47.20 ± 1.56	67.5 ± 3.25	68.87 ± 3.23
	VI SST	22.38 ± 2.53	26.17 ± 1.64	24.52 ± 8.84	30.07 ± 4.21
	VI Ice	22.93 ± 3.69	26.64 ± 2.49	7.98 ± 6.87	1.06 ± 1.11
	AUC	92.12 ± 1.85	95.50 ± 1.41	95.25 ± 1.67	95.94 ± 1.14
MaxEnt	VI Bare Rock	85.85 ± 0.89	77.37 ± 0.53	91.33 ± 0.61	41.5 ± 1.6
	VI SST	6.44 ± 0.81	13.07 ± 0.49	4.54 ± 0.86	44.74 ± 1.37
	VI Ice	7.71 ± 0.84	9.55 ± 0.51	4.12 ± 0.66	13.75 ± 0.69
	AUC	86.6 ± 0.14	91.46 ± 0.15	89.76 ± 0.31	90.71 ± 0.55

Supplemental Methods

Environmental variables

Similar to Cimino *et al.* 2013, from 1978-2011, sea ice concentration (SIC) was obtained from NSIDC's Nimbus-7 Scanning Multichannel Microwave Radiometer and the Defense Meteorological Satellite Program's Special Sensor Microwave/Imager. Sea surface temperature (SST) data was obtained from the NOAA Advanced Very High Resolution Radiometer (AVHRR) from 1981-2009 and NASA's MODIS Aqua satellite from 2010-2011. There was no discontinuity in the SST data when satellite platforms changed from AVHRR to MODIS Aqua[1]. Bare rock locations were obtained from Landsat 8; this data is included in the Antarctic Digital Database.

We used climate projections from GFDL climate version 2.1 (CM2.1) because it was considered to be one of four best models for gauging penguin habitat[2] and GFDL climate version 2.6 (CM2.6) is similar to CM2.1 but makes high-resolution projections of Antarctic coastal climate unlike more widely used models assessed by the Intergovernmental Panel on Climate Change (IPCC). GFDL-CM2.1 and CM2.6 are coupled atmosphere-ocean-land-sea ice global models. CM2.1 uses a nominal 1.0º grid spacing and has been widely used as part of the IPCC[3]. CM2.6 is a higher-resolution model that uses a nominal 0.10º grid spacing, with improved representation of the ocean mesoscale and is a more accurate simulation compared to the previous suite of Climate Models in version 2.0–Ocean[4-6]. Only CM2.1 included eddy parameterization in the ocean component.

Penguin chick-rearing habitat suitability models

We modeled the suitability of Adélie penguin chick-rearing habitats using two species distribution modeling approaches, a maximum entropy approach (MaxEnt version 3.3.3k[7]) and

generalized additive models (GAMs, BIOMOD package[8]). Both approaches are capable of fitting complex surfaces and non-linear relationships. MaxEnt uses presence-only locations and pseudo-absences from background data to compute the maximum entropy distribution. GAMs predicted habitat suitability using a binomial error structure and presence-absence (PA) locations. Although it is unadvised to use presence-only methods (MaxEnt) when PA data is available, we found it informative to compare results from MaxEnt and GAMs. We excluded duplicate presence records from our training dataset, which resulted from merging colony locations onto our large-scale grid. We fit MaxEnt models with default settings[1], and when necessary, increased the regularization parameter to smooth fitted relationships. When changing the regularization, we chose settings that limited complex fits through visual inspection of response curves and used a multiplier of 2.6[9]. The GAMs used smoothing splines as the smoother functions with 3 knots to avoid over-fitting.

For both modeling approaches, we used a cross-validation resampling procedure with four replicate runs that partitioned 75% of the penguin colonies into the fitting fold and 25% of the colonies into the left out fold[1]. This allowed for assessment of predictive performance on the held-out folds using the area under the receiver operating characteristics curve (AUC). The AUC is an indicator of the accuracy of the models, where 1 represents a model with perfect performance and 0.5 indicates a model that is no better than random[10]. In MaxEnt, jackknife tests were used to quantify which environmental predictors are contributing the most to fitting the model. For GAMs, we estimated the importance of each predictor variable as described by[11]. In all models, the species prevalence was set to 0.147, which is the true prevalence of Adélie penguins in the Southern Ocean on our polar stereographic grid. We compared MaxEnt and GAM predictions (Supplemental Fig. 25), fitted response functions (Supplemental Figs. 12, 26),

variable importance and AUC (Supplemental Table 2).

<u>Matching novel climate and chick-rearing habitat suitability to penguin colony locations</u>

From 1981-2010, we matched predicted trends in CRHS from MaxEnt and GAMs to colony locations and compared trends in CRHS in different Antarctic sectors. We also determined the number of years with novel climate for each coastline pixel, which was also matched to documented population trends. If novel climate occurred at a colony with a population status, we determined the main cause for that novel climate: warm SST, cool SST or high SIC (low SIC was not a category because the lowest SIC (zero sea ice) was documented). We used a nonparametric Kruskal–Wallis test to determine if there were significant differences between documented penguin population statuses and the number of years with novel climate. We also used a multiple comparison test after Kruskal–Wallis to determine if the number of years with novel climate differed between population groups. For future climate projections, we matched novel climate to current colony locations to understand how conditions at those colonies could change in the future.

<u>Supplemental Results</u>

To verify and understand the sensitivity of species distribution model predictions (Fig. 2), we tested different combinations of PA datasets (all = full dataset, Cont = only continental locations; Fig. 1) and used two species distribution model approaches (MaxEnt and GAMs) (Supplemental Fig. 25). Mean CRHS from respective GAM and MaxEnt models were highly correlated (Pearson correlations, $r > 0.95$, $p < 0.05$). We compared trends in CRHS from respective GAM and MaxEnt models and found ALL PA and Cont P/All A were highly correlated ($r > 0.90$, $p < 0.05$), Cont P/A were significantly correlated ($r = 0.71$, $p < 0.05$), and All P/Cont A had a lower, yet significant correlation ($r = 0.39$, $p < 0.05$). A noticeable difference

between MaxEnt and GAMs appears in the Cont P/A example in which MaxEnt produces results more similar to All P/A compared to the respective GAM. In general, the high correlations between MaxEnt and GAM predictions agree with other studies demonstrating the high correlation between presence-only MaxEnt and PA GAM results[12] but we also show that model results are more similar when PA data is complete. All models performed well (area under the curve (AUC) > 0.85, Supplemental Table 2) and predicted trends in suitability were higher at locations with available bare rock, which is necessary for nesting (Supplemental Fig. 27, 28). The trends in CRHS by Antarctic sector at present, absent, and no bare rock locations highlight how model predictions deviate based on given PA data (Supplemental Fig. 28). Predictions can be further explained by response curves (Supplemental Fig. 12, 26) and variable importance (Supplemental Table 2).

The WAP is a warmer environment compared to the continent (Fig. 1), and thus, represents a different environmental niche that Adélie penguins occupy. Models that did not include WAP absence or pseudo-absence data over-predicted mean and trends in CRHS in the WAP (ex. Cont P/A and more so for All P/Cont A, Supplemental Fig. 25). The response curves show a change in the right hand tail of the SST distribution when WAP absence data was excluded (Supplemental Fig. 12, 26). SST and SIC observations that were outside the range of the model training data, caused the models to extrapolate into novel climate. For All P/Cont A, excluding absence data along the warmer WAP resulted in higher suitability predictions for all WAP locations because the model has information that the penguin colonies are present within this SST and SIC range. Furthermore, losing absence data across part of the range (All P/Cont A) appears more harmful than losing presence data across part of the range (Cont P/All A). This can be seen in the mean CRHS and trends in CRHS in which CRHS did not substantially change

when WAP presence data was excluded (All P/A vs. Cont P/All A) while much higher CRHS was seen along the WAP when WAP absence data is excluded (All P/A vs. All P/Cont A) (Supplemental Fig. 25 b,d). This highlights the importance of having absence data throughout the entire environmental range that a species occupies and the sensitivity of species distribution models to input PA data (also noted by[9]). Overall, models varied more based on treatment of PA data than modeling method (as concluded by[9]).

Similar model output using true absences and pseudo-absences indicates the MaxEnt models and GAMs produced valid results. We are not suggesting that other studies use MaxEnt when PA data are available (also see[13]) but rather, in our case MaxEnt performed similar to GAMs. We also suggest using caution when solely using the AUC as a metric for determining model performance. In our study, all of our models had an AUC > 0.85 but we know the models extrapolated into novel climate, especially when PA data was incomplete. In ecology, it is rare to have true PA data as in this study, which makes it important to evaluate the distribution of your PA data, and response curves, in relation to your projection environment.

References

1. Cimino, M. A., Fraser, W. R., Irwin, A. J. & Oliver, M. J. Satellite data identify decadal trends in the quality of *Pygoscelis* penguin chick-rearing habitat. *Glob. Change Biol.* **19**, 136-148 (2013).
2. Ainley, D. *et al*. Antarctic penguin response to habitat change as Earth's troposphere reaches 2°C above preindustrial levels. *Ecol. Monogr.* **80**, 49-66 (2010).
3. Delworth, *et al*. GFDL's CM2 global coupled climate models. Part I: Formulation and simulation characteristics. *J. Climate* **19**, 643-674 (2006).
4. Winton, M. *et al*. Has coarse ocean resolution biased simulations of transient climate sensitivity. *Geophys. Res. Lett.* **41**, 8522-8529 (2014).
5. Griffies, S. M. *et al*. Impacts on ocean heat from transient mesoscale eddies in a hierarchy of climate models. *J. Climate* **28**, 952-977 (2015).
6. Delworth, T. L. *et al*. Simulated climate and climate change in the GFDL CM2. 5 high-resolution coupled climate model. *J. Climate* **25**, 2755-2781 (2012).
7. Phillips, S. J., Anderson, R. P. & Schapire, R. E. Maximum entropy modeling of species geographic distributions. *Ecol. Model.* **190**, 231-259 (2006).
8. Thuiller, W. BIOMOD: optimizing predictions of species distributions and projecting potential future shifts under global change. *Glob. Change Biol.* **9**, 1353-1362 (2003).
9. Elith, J., Kearney, M. & Phillips, S. The art of modelling range-shifting species. *Methods Ecol. Evol.* **1**, 330-342 (2010).
10. Hosmer, D. W. & Lemeshow, S. *Applied logistic regression* (Wiley-Interscience, New York, 2000).
11. Thuiller, W., Lafourcade, B., Engler, R. & Araújo, M. B. BIOMOD–a platform for

ensemble forecasting of species distributions. *Ecography* **32**, 369-373 (2009).

12. Hijmans, R. J. & Graham, C. H. The ability of climate envelope models to predict the effect of climate change on species distributions. *Glob. Change Biol.* **12**, 2272-2281 (2006).

13. Guillera-Arroita, G. & Lahoz-Monfort, J. J. Maxent is not a presence–absence method: a comment on Thibaud et al. *Methods Ecol. Evol.* **5**, 1192-1197 (2014).
